# Fourteen New Species of Foliar *Colletotrichum* Associated with the Invasive Plant *Ageratina* *adenophora* and Surrounding Crops

**DOI:** 10.3390/jof8020185

**Published:** 2022-02-13

**Authors:** Zefen Yu, Xinwei Jiang, Hua Zheng, Hanbo Zhang, Min Qiao

**Affiliations:** 1 Laboratory for Conservation and Utilization of Bio-Resources, Yunnan University, Kunming 650091, China; zfyu@ynu.edu.cn (Z.Y.); Jiangxinwei@mail.ynu.edu.cn (X.J.); zhenghua@mail.ynu.edu.cn (H.Z.); 2 School of Life Sciences, Yunnan University, Kunming 650091, China

**Keywords:** endophytes, morphology, multi-locus, novel species, taxonomy

## Abstract

*Ageratina adenophora* is one of the most invasive weeds in China. Following an outbreak in Yunnan in the 1960s, *A. adenophora* has been spreading in Southwest China at tremendous speed. Previous research indicated *A. adenophora* contained many *Colletotrichum* species as endophytes. In this study, we investigated the diversity of *Colletotrichum* in healthy and diseased leaves of the invasive plant *A. adenophora* and several surrounding crops in Yunnan, Guangxi, and Guizhou provinces in China, and obtained over 1000 *Colletotrichum* strains. After preliminary delimitation using the internal transcribed spacer region (ITS) sequences, 44 representative strains were selected for further study. Their phylogenetic positions were determined by phylogenetic analyses using combined sequences of ITS, actin (*ACT*), chitin synthase (*CHS*-1), glyceraldehyde-3-phosphate dehydrogenase (*GAPDH*), and beta-tubulin (*TUB*2). Combined with morphological characteristics, 14 new *Colletotrichum* species were named as *C. adenophorae*, *C. analogum*, *C. cangyuanense*, *C. dimorphum*, *C. gracile*, *C. nanhuaense*, *C. nullisetosum*, *C. oblongisporum*, *C. parvisporum*, *C. robustum*, *C. simulanticitri*, *C. speciosum*, *C. subhenanense*, and *C. yunajiangense*.

## 1. Introduction

Foliar fungal endophytes are defined as fungi that can colonize the internal plant leaf structure without causing apparent harm to the aerial parts of the host [1]. Foliar tissues of all plant species examined to date harbor at least one and, more commonly, a wide diversity of fungi lurking within their plant tissues [2]. These fungi phylogenetically span at least four phyla [3] and are categorized into two groups based on their manner of transmission: vertically transmitted endophytes (VTEs, e.g., *Neotyphodium* Glenn et al.) that inhabit temperate grasses (*Lolium* L.) and are transmitted from the mother plant [4] and horizontally transmitted endophytes (HTEs) transmitted mostly via spores from plant to plant via rain, wind, local leaf litter, or insect vectors [5,6]. HTEs are so common that the foliar tissues of all plant species examined thus far harbor at least one fungus [2]. Although the majority of these endophytic fungi are localized infections by either latent pathogens or dormant saprophytes [2], in many cases endophytes may promote host plant growth, improve nutrient supply and protect plants from both biotic and abiotic stresses [7,8]. Because a plant can be colonized by endophytic fungi that originate from a neighbor or by spores that persist in the local environment, it is not surprising that endophyte communities found in the same plant host species can show striking geographical differences [6].

Non-native (alien, exotic, introduced) species are species whose presence in a region is attributable to human actions that enabled them to overcome fundamentally biogeographic barriers. Only a fraction of non-native species become invasive; they are defined as species that become naturalized, that is, sustain self-replacing populations over several life cycles and have the potential to spread over long distances and dominate local communities [9]. When invasive plants are introduced into a new area, they will likely develop a novel relationship with the local microorganisms and generate impacts on ecosystems [10,11]. In many cases, these novel relationships rather than the plant itself determine the invasiveness of an exotic plant in an introduced range [12]. Many works have investigated the belowground microbes (soils) associated with invasive plants and their roles [13,14,15]. Invasive plants encounter foliar fungal endophytes; however, this community of endophytes is investigated less often. Until now, only a few reports have evaluated these fungi. Genetic analysis of endophytic fungi found in *Centaurea stoebe* L. in both its native and invaded ranges showed that 85% occurred in only one of the two ranges [16]. In Europe, the native range, the most common endophyte was a haplotype of *Alternaria alternata* (Fr.) Keissl. In the invaded range, no haplotype was dominant, and many were novel. Some fungal endophytes appear to have been introduced with *C. stoebe*, but the invader also acquired new endophytes after introduction [16]. The most common OTU corresponded most closely to *Sarocladium strictum* (W. Gams) Summerb. and comprised 25% of all fungal isolates. The functional role of the isolated endophytes is not yet known, but one genus isolated here (*Stagonospora* (Sacc.) Sacc.) has been reported to enhance *Phragmites* growth. Some of the fungal endophytes isolated here may correspond to latent pathogens (e.g., OTU 2) that either have not expressed their pathogenic phase [17] or are not pathogenic to non-native *Phragmites* [18,19]. Non-native *P. australis* (Cav.) Trin. ex Steud. decreases biodiversity and produces dense stands in North America. We surveyed the endophyte communities in the stems, leaves, and roots of collections of *P. australis* obtained from two sites with a low and high salt concentration to determine differences in endophyte composition and assess differences in the functional roles of microbes in plants from both sites. The high salt endophyte community showed higher resistance to zinc, mercury, and salt stress compared to fungal species from the low salt site. These endophytes also showed a greater propensity for growth promotion of rice seedlings (a model species) under salt stress. The results of this study were consistent with the ‘habitat-adapted symbiosis hypothesis’ that holds that endophytic microbes may help plants adapt to extreme habitats. The capacity of *P. australis* to establish symbiotic relationships with diverse endophytic microbes that enhance its tolerance to abiotic stresses could be a factor contributing to its invasiveness in saline environments [20].

Generally, endophytes are beneficial to the invasion of the host (there are also contrary examples, e.g., [21,22]). For example, the invasive forb species *C. stoebe* has allelopathic effects on native species that are significantly enhanced when it is infected by a fungal endophyte closely related to *A. alternata* [23]. The competitive effects of *C. stoebe* on grass species native to North America were enhanced by both *Alternaria* endophytes, but not on native European grasses [24]. Similarly, some fungal endophytes isolated from *C. stoebe* seeds caused significant declines in germination of *Festuca idahoensis* Elmer, a North American grass competitor [25]. Recently, fungi isolated from an invasive plant *Vincetoxicum rossicum* (Kleopow) Barbar. were proven to have differential growth effects between non-native invasive and native plants, which may be important for mediating plant community structure and the persistence of invasive plants [26].

*Ageratina adenophora* (Sprengel) King and Robinson (syn. *Eupatorium adenophorum* Spreng.) (Asteraceae), also known as Crofton weed or Mexican Devil weed, originates in Mexico and is an invasive weed in several countries worldwide, including Australia, China, Hawaii, India, New Zealand and South Africa [27,28]. It is one of the most invasive weeds in China. Following an outbreak in Yunnan in the 1960s, *A. adenophora* has been spreading north- and eastward through Guizhou, Sichuan, and Guangxi Provinces in Southwest China at approximately 20 km/year [29]. To determine if *A. adenophora* could benefit from the fungal endophytes accumulated in the introduced range, our team previously reported that *A. adenophora* actually contained diverse fungal endophytes; in particular, *Colletotrichum* Corda comprised 30% of total isolated strains [30]. However, the foliar *Colletotrichum* generally adversely affect leaf development in *A. adenophora* and may be a weak latent foliar pathogen [31]. Our further disease experiments verified that some of these *Colletotrichum* are pathogenic on local native plants [32]. Indeed, *Colletotrichum* species are among the most commonly occurring pathogens and foliar endophytes of terrestrial plants [33,34]. The *Colletotrichum* genus was recently included in the list of the 10 most important plant pathogenic fungi, based on perceived scientific and economic importance [35]. The genus *Colletotrichum* comprises ~600 species attacking over 3200 species of monocot and dicot plants [36]. In addition, many *Colletotrichum* species are latent plant pathogens, these species being essentially endophytes, epiphytes, or saprobes, switching to a pathogenic lifestyle when host plants are stressed or in postharvest storage [37,38]. It is common to find multiple *Colletotrichum* species infecting a single host [39]. Although it has also been shown that particular *Colletotrichum* endophytes confer protective benefits on cacao hosts by reducing disease incidence and damage caused by other plant pathogens [40,41,42], in this case, there is a possible ecological risk of disease transmission to local plants driven by *A. adenophora* invasion.

Our current understanding of *Colletotrichum* species is largely restricted to populations affecting crop or ornamental plants, with poor knowledge of non-agricultural crops [34]. Higgins et al. [43] studied *Colletotrichum* endophytes from grass and non-grass hosts in a tropical forest in Panama, recovering some genetically distinct taxa via direct sequence from surface-sterilized grass tissue that were not detected using cultural methods. They also observed that many taxa were detected from more than one grass host genus, corroborating observations by Lu et al. [44] and Arnold and Lutzoni [3] that the commonest tropical endophytes appear to be host generalists. All the studies of *Colletotrichum* associated with non-crop plants detailed above demonstrated considerable diversity of taxa. Despite preliminary evidence of less host specificity in native tropical forest ecosystems compared with managed environments, the sheer number of habitats (in the form of leaves, fruits, etc.) that remain unsampled indicates the likelihood that overall species-level diversity of the genus is still significantly under-represented. Studies of *Colletotrichum* from wild plants are likely to be particularly fruitful. It would be presumptuous even to speculate that the overall systematic framework for the genus cannot be improved [34]. Several endophyte taxa isolated from cacao in Panama by Rojas et al. [45] were thought to comprise part of the background endophytic community in the Panamanian forest ecosystem [45].

Previously, the identification of *Colletotrichum* species was based on morphological characteristics. Cai et al. [46] found that such morphological identification of *Colletotrichum* species depended on the experimental methods used, which caused the taxonomy and nomenclature to be inconsistent. Recently, Cai et al. recommended a polyphasic approach for accurate identification of *Colletotrichum* species using multi-locus phylogeny coupled with morphological data. Using this approach, many *Colletotrichum* strains have been successfully identified and epitypified. This increased understanding of *Colletotrichum* species can increase the effectiveness of plant disease control interventions [47].

In the present study, we investigated the diversity of *Colletotrichum* in healthy and diseased leaves of invasive plant *A. adenophora* and several surrounding crops in Yunnan, Guangxi, and Guizhou Provinces in China, and obtained over 1000 *Colletotrichum* strains. Of these, 44 representative strains were selected for further study, including five-locus phylogenetic analyses and morphological characteristics. Finally, these strains were assigned to 26 *Colletotrichum* species, including 12 known *Colletotrichum* species and 14 new species. The results of this study will contribute to the knowledge on the diversity and phylogeny of *Colletotrichum* associated with an invasive plant and surrounding crops. Because the *Colletotrichum* genus is one of the most important pathogenic fungi [35], our finding is also valuable for understanding the possible disease risk driven by invasive plants in the invaded ecosystem.

## 2. Materials and Methods

### 2.1. Sample Collection and Isolation of Fungi

The *Colletotrichum* strains used in this study were collected from healthy and diseased leaves of the invasive plant *Ageratina adenophora*, as well as diseased leaves of native plants, from 2012 to 2018, using leaf fragment incubation [32,48]. Briefly, for the isolation of leaf spot fungi, healthy leaf tissues and the margins of diseased tissues of each leaf spot were cut into 6 mm^2^ sections and surface sterilized as follows. These sections underwent initial immersion for 2 min in 0.5% sodium hypochlorite, followed by 1 min in sterile distilled water, 2 min in 75% ethanol, and, finally, 1 min in sterile distilled water. The disinfected fragments were then plated onto potato dextrose agar (PDA; 200 g potato, 20 g glucose, 18 g agar, 1 L distilled water) and incubated at ambient temperature for 6–8 days or until mycelia growing from the leaf fragments were observed. In total, over 1000 *Colletotrichum* strains were collected and we selected 44 representative strains for further study.

The pure cultures and dried cultures were deposited in the Herbarium of the Laboratory for Conservation and Utilization of Bio-resources, Yunnan University, Kunming, Yunnan, China (YMF) and the China General Microbiological Culture Collection Center (CGMCC).

### 2.2. Morphological Characterization

The growth rates of *Colletotrichum* isolates were determined on four common media: cornmeal dextrose agar (CMA; 20 g cornmeal, 18 g agar, 1 L distilled water), low nutrient agar (SNA; 1 g KH_2_PO_4_, 1 g KNO_3_, 0.5 g KCl, 0.5 g MgSO_4_, 0.2 g sucrose, 0.2 g glucose, 18 g agar, 1 L distilled water), malt extract agar (MEA; 30 g malt powder, 3 g peptone, 18 g agar, 1 L distilled water), and PDA, respectively. Six-millimeter plugs of actively growing mycelia were inoculated onto these media (20 mL per plate); these cultures were incubated at 25 °C for 7 days. During this time, the colony diameters and characteristics, such as colony appearance, color, and sporogony, on the four media were recorded. Moreover, microscopic characteristics were identified by photographing with a BX51 microscope connected to a DP Controller digital camera. Microscopic characteristics were created from cultures growing on CMA or SNA at 25 °C.

### 2.3. DNA Extraction, PCR Amplification, and Sequencing

Genomic DNA was extracted from the fresh mycelium harvested from PDA plates after 4 days, as described by Wang and Zhuang [49]. Five loci, the nuc rDNA internal transcribed spacers (ITS), actin (*ACT*), chitin synthase (*CHS*-1), glyceraldehyde-3-phosphate dehydrogenase (*GAPDH*), and beta-tubulin (*TUB*2), were amplified with the following primer pairs, ITS4 and ITS5 for ITS [50],GDF1 and GDR1 for *GAPDH* [51], ACT-512F and ACT-783R for *ACT* [52], CHS-79F and CHS-354R for *CHS*-1 [52], and Bt2a and Bt2b for *TUB*2 [53]. The ITS sequence fragment was used for original identification; then the ITS together with *GAPDH*, *CHS*-1, *ACT* and *TUB*2 were used to construct a phylogenetic tree of the genus *Colletotrichum*. A PCR reaction was conducted in a 25 μL reaction volume, and the components were as follows: 1 μL DNA template, 1 μL forward 10 μM primer, 1 μL reverse 10 μM primer, 12.5 μL T5 Super PCR Mix (containing Taq polymerase, dNTP and Mg^2+^, Beijing TisingKe Biotech Co., Ltd., Beijing, China), 5 μL enhance buffer and 4.5 μL sterile water. PCR reactions were run in an Eppendorf Mastercycler ep with related PCR thermal cycle programs described by Zhuang and Chen [54].

PCR products were firstly checked by performing 1.5% agarose gel electrophoresis stained with ethidium bromide, then purified using the PCR product purification kit (Biocolor BioScience and Technology Co., Shanghai, China), and finally sent for both forward and reverse sequencing on an ABI 3730 XL DNA sequencer (Applied Biosystems, Foster City, CA, USA) with the same primers at Tsingke Biotechnology Co., Ltd. (Kunming, China). Then, the forward and reverse sequences were assembled to obtain a consensus sequence using DNAMAN v. 9.0. These newly generated sequences were deposited in the GenBank database, and the accession numbers from this study in the National Center for Biotechnology Information (NCBI) are listed in Supplementary Table S1.

### 2.4. Multigene Phylogenetic Analyses

The phylogenetic tree was constructed with five loci *ACT*, *CHS*-1, *GAPDH*, *TUB*2, and ITS to identify these *Colletotrichum* isolates to the species level. Additional reference species sequences were selected based on recent studies on *Colletotrichum* species [55,56], referring to 14 species complexes and 13 singleton species. Both the reference sequences and new generated sequences in this study are listed in Supplementary Table S1. Multiple sequence alignments for the five loci were constructed and carried out using Clustalx v. 1.83 [57] with the default settings. The phylogeny was developed based on maximum-likelihood for all individual loci. Then, five datasets of *ACT*, *CHS*-1, *GAPDH*, *TUB*2, and ITS of *Colletotrichum* spp. were manually adjusted and linked through BioEdit v. 7.0 [58]. Of these, gap adjustments were performed and ambiguously aligned regions were also excluded. The final concatenated sequence matrix (Fasta file) generated by BioEdit v. 7.0 contained 2119 characters with gaps, *ACT* with 310 sites, *CHS* with 299 sites, *GAPDH* with 363 sites, *TUB*2 with 532 sites, and ITS with 615 sites. Gaps in the concatenated dataset were treated as missing data in maximum-likelihood (ML) trees and Bayesian inference (BI) methods.

ML analysis was performed using RAxML v. 8.2.10 [59] with the PHY files, which were generated using CLUSTAL_X v. 1.83 [57]. Final ML searches were conducted using the GTR-GAMMA model. Maximum likelihood bootstrap proportions (MLBP) were computed with 1000 replicates and were given above each node of the phylogenetic tree when it was equal to or greater than 70. For BI analysis, a Markov Chain Monte Carlo (MCMC) algorithm was used to generate phylogenetic trees using MrBayes v. 3.1.2 [60]. The concatenated dataset was converted to a NEXUS file with MEGA6 [61], then the Akaike information criterion (AIC) was implemented by using jModelTest v. 2.0 [62] to select the best fit models of nucleotide substitution. GTR+I+G+F was selected as the best fit model for the concatenated dataset. Two independent analyses with four chains were run for 4,000,000 generations sampling every 1000th generation, halting the analyses when the average standard deviation of split frequencies was less than 0.01. The first 25% of the tree generations were discarded as the burn-in phase of the analyses, and the Bayesian inference posterior probability (BIPP) was obtained from the remaining trees. Trees generated in this study were viewed with TreeView v. 1.6.6 [63], and the final topology of phylogenetic trees was based on maximum likelihood analysis, with MLBP greater than 70 and BIPP greater than 0.9, as shown at the nodes.

## 3. Results

### 3.1. Multi-Locus Phylogeny

**Figure 1:**
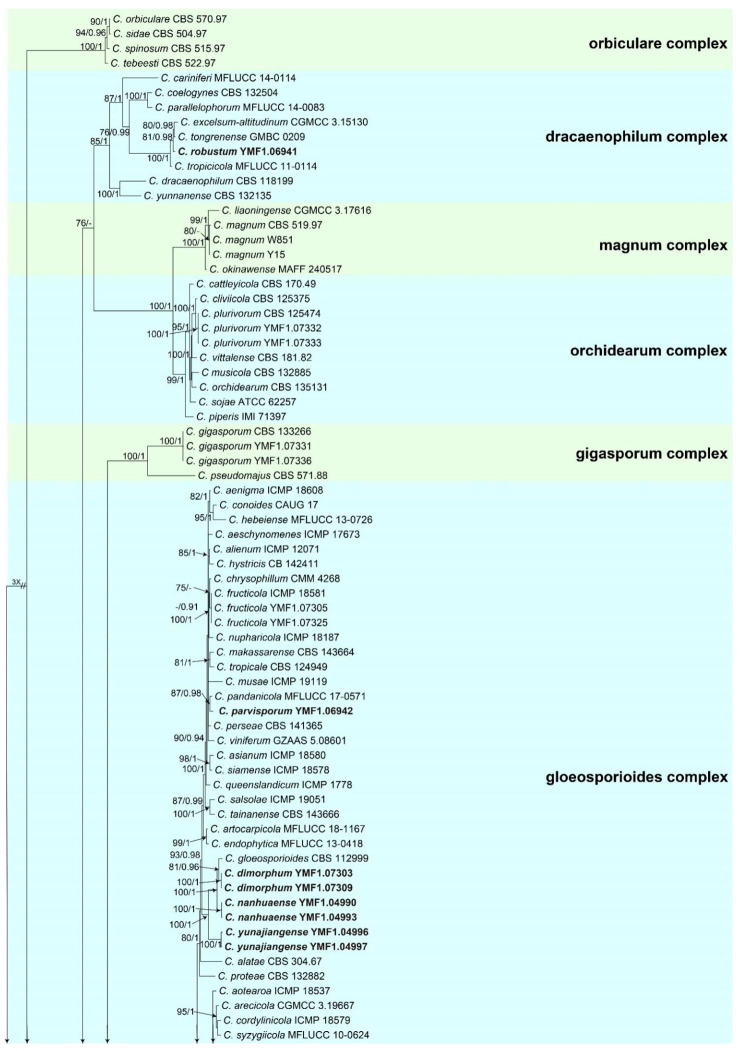

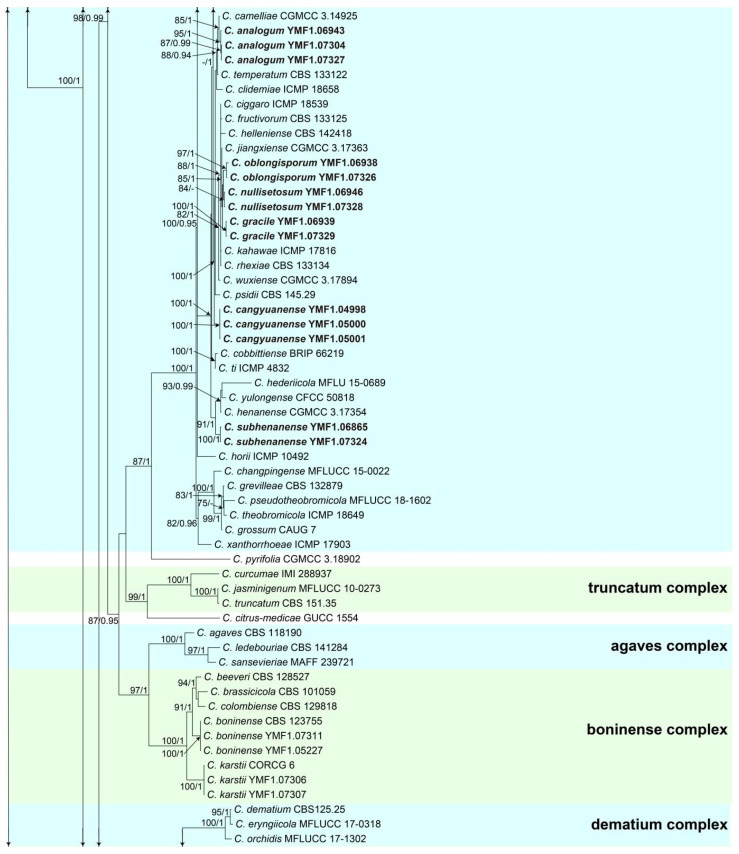

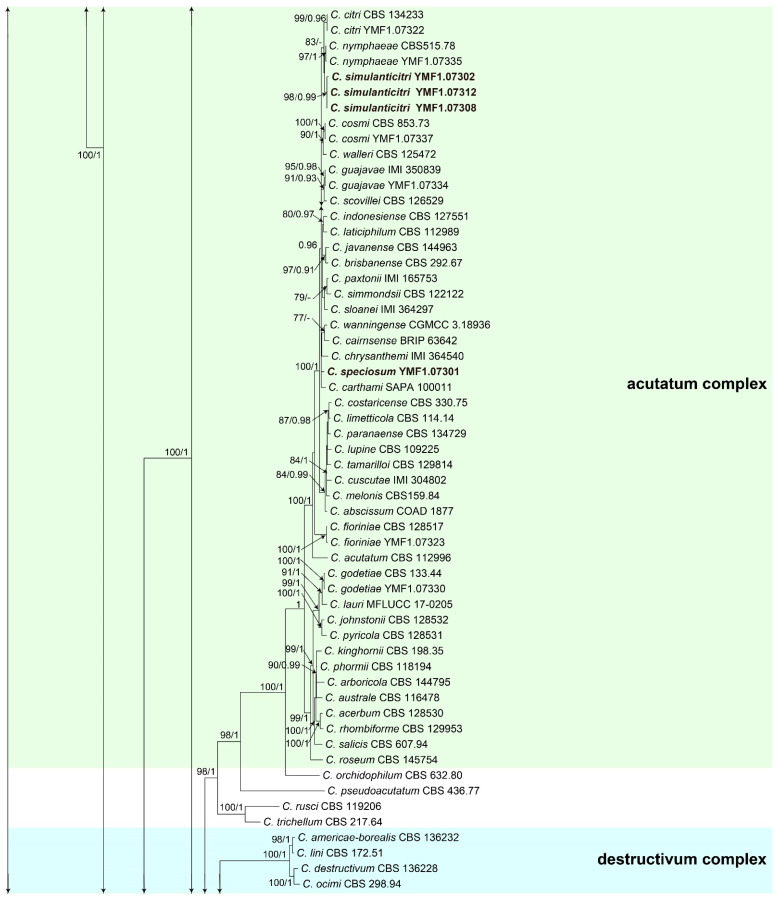

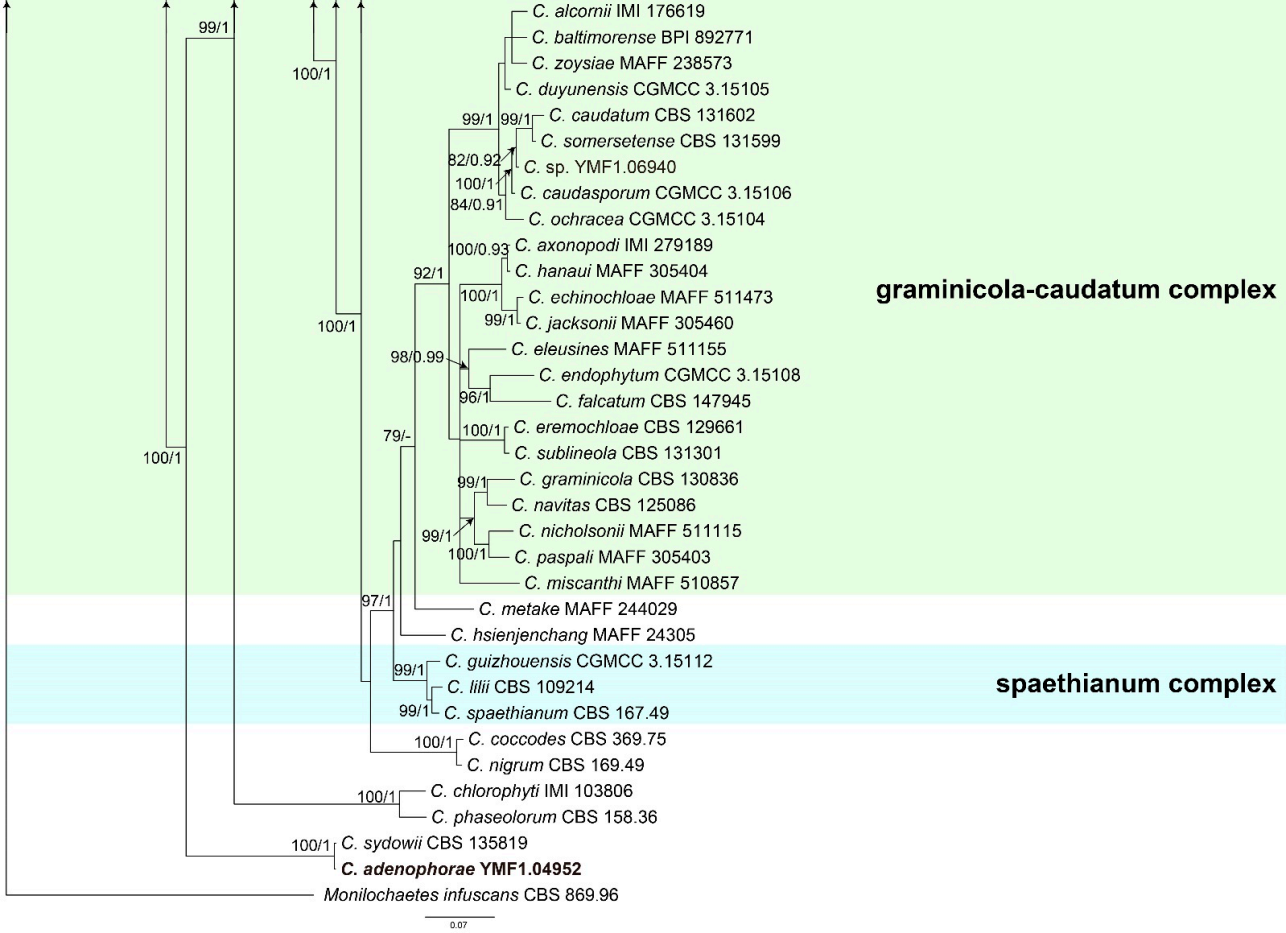
Phylogenic tree generated by the maximum likelihood analysis using combined sequences of ACT, CHS-1, GAPDH, ITS, and TUB2 loci of the genus *Colletotrichum*. Bootstrap values ≥ 70 (**left**) and Bayesian posterior probability values ≥ 0.9 (**right**) are indicated at nodes (MLBP/BIBP). *Monilochaetes infuscans* CBS 869.96 was used as the outgroup. Novel species generated in this study are indicated in black bold.

All *Colletotrichum* spp. isolates were first identified at genus level based on their ITS sequences. To further identify these isolates at species level, we conducted multi-locus phylogenetic analysis referring to 2119 characters and 220 sequences. For the five-locus (*CHS*-1, *GAPDH*, ITS, *ACT*, and *TUB*2) phylogenetic analysis of the genus *Colletotrichum*, 173 reference sequences obtained from GenBank and our selected 46 representative sequences of *Colletotrichum* isolates were used in this study. *Monilochaetes infuscans* Harter CBS 869.96 was selected as the outgroup according to recent publications [55,56]. When the five individual sequence datasets did not show any conflicts in the tree topologies based on preliminary ML analyses, the phylogeny was developed based on both ML and BI for the combined multi-locus analyses (Figure 1).

The results of this analysis revealed that 46 isolates were distributed in eight species complex clades, which were, respectively, the *C. dracaenophilum* species complex, the *C. magnum* species complex, the *C. orchidearum* species complex, the *C. gigasporum* species complex, the *C. gloeosporioides* species complex, the *C. boninense* species complex, the *C. acutatum* species complex, and the *C. graminicola-caudatum* species complex clades, and one isolate cluster with the singleton species *C. sydowii* CBS 135819.

For the isolates in the *C. dracaenophilum* species complex, one isolate clustered with *C. excelsum-altitudinum* and *C. tongrenense* (MLBP 81/ BIBP 0.98); we designated the isolate as *C. robustum*.

For the isolates in the *C. magnum* species complex, two isolates clustered with the *C. magnum* clade (MLBP 80) corresponding to the known species *C. magnum*. For the isolates in the *C. orchidearum* species complex, two isolates were grouped with the *C. plurivorum* clade (100/1) and identified as the known species *C. plurivorum*.

For the isolates in the *C. gigasporum* species complex, two isolates clustered with the *C. gigasporum* clade (100/1) corresponding to *C. gigasporum*.

For the isolates in the *C. gloeosporioides* species complex, 23 isolates clustered in 11 clades corresponding to *C. fructicola* (100/1), *C. parvisporum* (87/0.98), *C. dimorphum* (100/1), *C.* *nanhuaense* (100/1), *C.* *yunajiangense* (100/1), *C. analogum* (95/1), *C. oblongisporum* (95/1), *C. nullisetosum* (MLBP 84), *C. gracile* (100/1), *C.* *cangyuanense* (100/1), and *C.* *subhenanense* (100/1).

For the isolates in the *C. boninense* species complex, four isolates were distributed in two clades and identified as two known species *C. boninense* (100/1) and *C. karstii* (100/1). For the isolates in the *C. acutatum* species complex, ten isolates were distributed in eight clades corresponding to *C. citri* (99/0.96), *C. nymphaeae* (97/1), *C. simulanticitri* (98/0.99), *C. cosmi* (100/1), *C. guajavae* (95/0.98), *C. speciosum* (100/1), *C. fioriniae* (100/1), and *C. godetiae* (100/1).

In addition, one isolate clustered with the singleton species *C. sydowii* (100/1), designated as *C. adenophorae*.

### 3.2. Taxonomy

**Figure 2:**
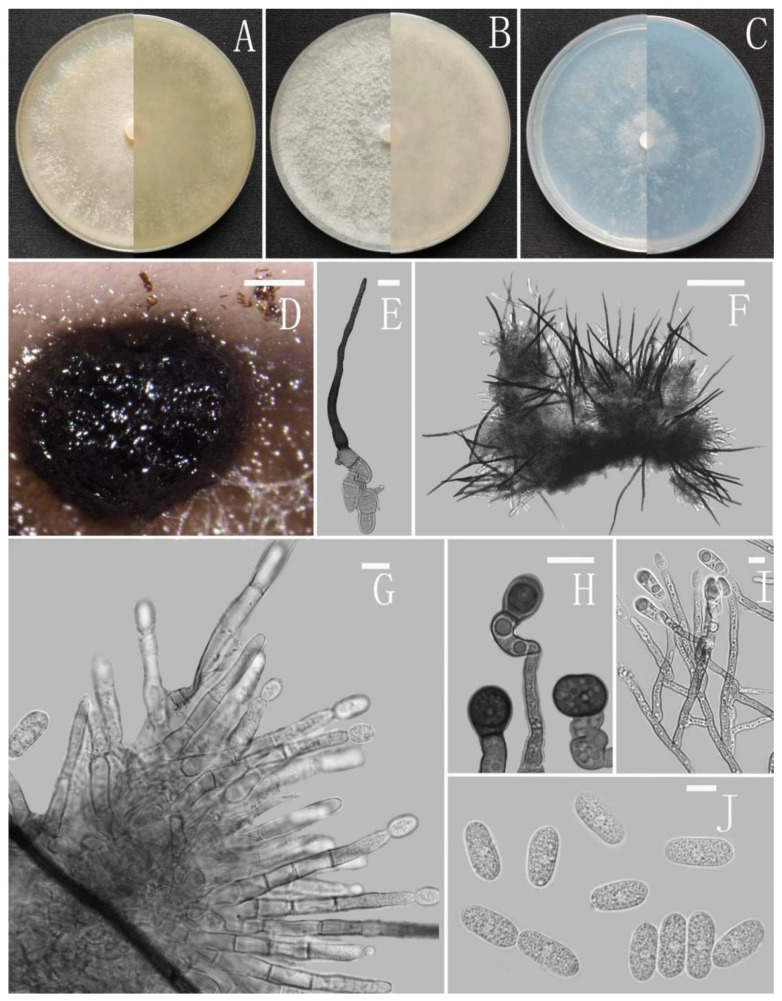
*Colletotrichum adenophorae* (YMF 1.04952). (**A**–**C**) Colonies on media above and below after 7 days at 25 °C ((**A**) MEA; (**B**) PDA; (**C**) SNA). (**D**) Conidiomata. (**E**,**F**) Setae. (**G**) Conidiophores and Conidia. (**H**) Appressoria. (**I**) Conidiophores. (**J**) Conidia. Scale bars: (**D**) = 100 μm, (**E**) = 10 μm, (**F**) = 50 μm, (**G**–**J**) = 10 μm.

*Colletotrichum adenophorae* Z.F. Yu (Figure 2).

MycoBank Number: MB842546.

Etymology: The species epithet refers to the host plant *Ageratina adenophora*.

Type: China, Yunnan province, Lancang county, isolated as an endophyte from *Ageratina adenophora*, August 2016, Han-Bo Zhang. Holotype YMF 1.04952, Ex-type culture CGMCC 3.18952.

Sexual morph not observed. Asexual morph on CMA. Conidiomata rudimentary, dark olivaceous brown. Setae and conidiophores formed directly on hyphae. Setae abundant, cylindrical, straight or slightly flexuous, dark brown to black, 4–6-septate, base cylindrical to slightly inflated, tip acute to rounded, 54–105 μm long. Conidiophores hyaline to pale brown, smooth-walled, septate, unbranched. Conidiogenous cells hyaline, cylindrical, smooth-walled, collarette absent. Conidia straightly cylindrical, oblong to narrowly ovoid, obtuse at the base, rounded at the apex, contents granular, 18–22.5 × 5.5–7.5 μm, mean ± SD = 20.2 ± 1.1 × 6.2 ± 0.4 μm, l/w ratio = 3.3. Appressoria dark brown, irregular, margin sometimes lobed, 6.5–14 × 5.5–12 μm.

Culture characteristics: Colonies on MEA flat with entire margin, occupied the whole plate in 7 days at 25 °C, surface buff, partly covered with a short, felty, whitish aerial mycelium, reverse medium buff to pale honey. Colonies on PDA flat with entire margin, occupied the whole plate in 7 days at 25 °C, aerial mycelium fluffy, flocculent, white in edges, reverse white to light yellow. Colonies on SNA flat with entire margin, occupied the whole plate in 7 days at 25 °C, covered with thin, whitish aerial mycelium, producing pale gray conidiomata in 2 weeks, reverse same colors. Colonies on CMA reaching 59–60 mm diameter in 7 days at 25 °C, aerial mycelium fluffy, flocculent, white, reverse same colors.

Notes: *Colletotrichum adenophorae* is phylogenetically clustered with the singleton species *C. sydowii* Damm. However, *C. adenophorae* is easily distinguished from *C. sydowii* in morphology. For instance, *C. sydowii* has smaller conidia [(14–)15.5–18.5(–20.5) × 5–6 vs. 18.0–22.5 × 5.5–7.5 μm] and less lobate appressoria [64].

**Figure 3:**
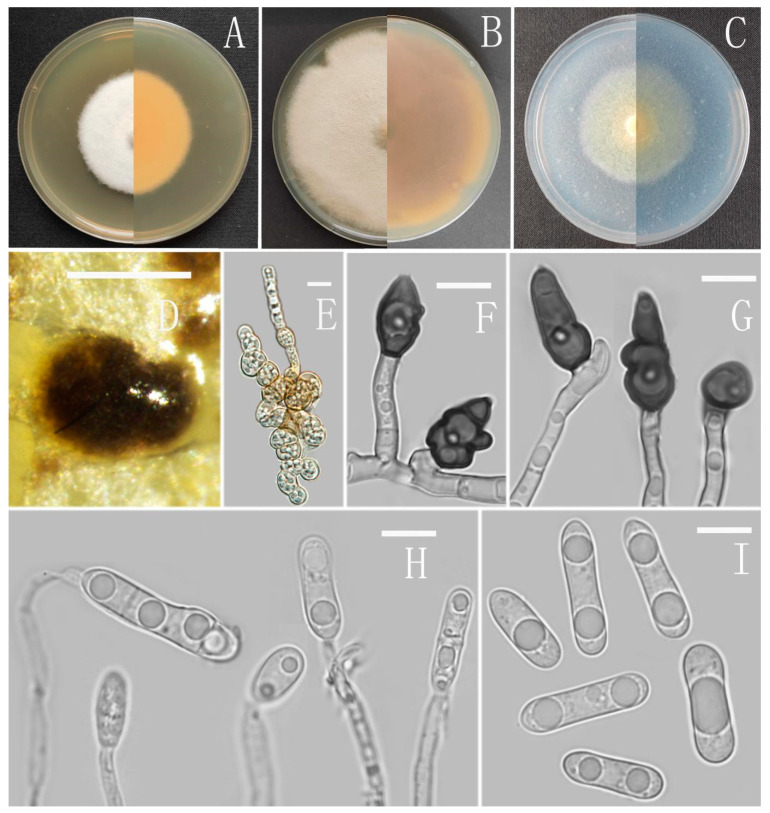
*Colletotrichum analogum* (YMF 1.06943). (**A**–**C**) Colonies on media above and below after 7 days at 25 °C ((**A**) MEA; (**B**) PDA; (**C**) SNA). (**D**) Conidiomata. (**E**) Chlamydospores. (**F**,**G**) Appressoria. (**H**) Conidiophores. (**I**) Conidia. Scale bars: (**D**) = 100 μm, (**E**–**I**) = 10 μm.

*Colletotrichum analogum* Z.F. Yu (Figure 3).

MycoBank Number: MB842547.

Etymology: The species epithet is based on the similarity or comparability with *Colletotrichum clidemiae*.

Type: China, Yunnan province, Ning’er county, from disease spot of *Ageratina adenophora*, August 2017, Han-Bo Zhang. Holotype YMF 1.06943, Ex-type culture CGMCC3.16079.

Sexual morph not observed. Asexual morph on CMA. Conidiomata rudimentary. Setae not observed. Chlamydospores pale yellow, 6.2–8.7 × 5.1–6.8 μm. Conidiophores directly formed on hyphae, hyaline, smooth-walled, 0–1-septate, unbranched. Conidiogenous cells hyaline, cylindrical, smooth-walled, collarette inconspicuous. Conidia cylindrical to clavate, unicellular, straight and slender, obtuse, subtruncate at the base, rounded at the apex, 15–20 × 5–6.5 μm, mean ± SD = 17.6 ± 1.4 × 5.5 ± 0.5 μm, l/w ratio = 3.2. Appressoria dark brown, septate, single or in small groups, irregular in outline, margin sometimes lobed, 9–18 × 6–10 μm.

Culture characteristics: Colonies on MEA flat with entire margin, reaching 48–50 mm diameter in 7 days at 25 °C, aerial mycelium fluffy, milky white, reverse same colors. Colonies on PDA reaching 84–88 mm diameter in 7 days at 25 °C, aerial mycelium dense, cottony, gray in the center and white in the margin, reverse grey to saffron with brown pigmentation ring. Colonies on SNA flat with entire margin, reaching 49–52 mm diameter in 7 days at 25 °C, medium buff to pale honey in the center, aerial mycelium fluffy, flocculent, pale yellow, white in edges, reverse buff to pale honey. Colonies on CMA reaching 39–40 mm diameter in 7 days at 25 °C, towards the margin honey to buff, partly covered with short felty whitish aerial mycelium, reverse olivaceous buff to olivaceous gray.

Additional specimens examined: China, Yunnan province, Ning’er county, from disease spot of *Ageratina adenophora*, August 2017, Han-Bo Zhang, living cultures YMF 1.07304, YMF1.07327.

Notes: Three strains of *Colletotrichum analogum* formed a sole clade in the gloeosporioides species complex, and the clade is close to *C. camelliae* Massee. Morphologically, *C. analogum* differs from *C. camelliae* by having narrowly cylindrical conidia, while those of *C. camelliae* are slightly constricted in the middle [65].

**Figure 4:**
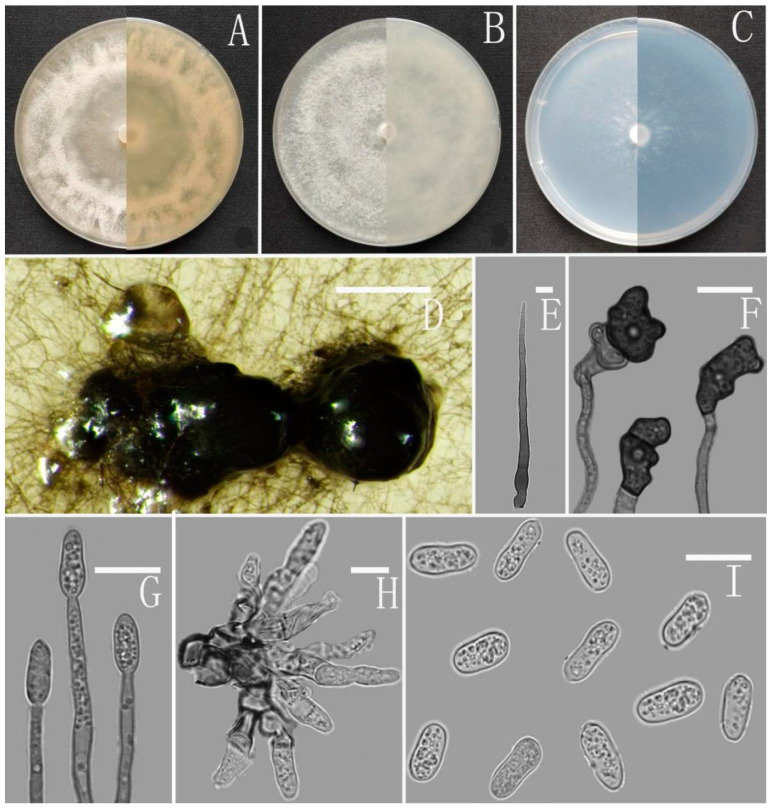
*Colletotrichum cangyuanense* (YMF 1.05001). (**A**–**C**) Colonies on media above and below after 7 days at 25 °C ((**A**) MEA; (**B**) PDA; (**C**) SNA). (**D**) Conidiomata. (**E**) Setae. (**F**) Appressoria. (**G**) Conidiophores. (**H**) Conidiophores and Conidia. (**I**) Conidia. Scale bars: (**D**) = 100 μm, (**E**–**I**) = 10 μm.

*Colletotrichum cangyuanense* Z.F. Yu (Figure 4).

MycoBank Number: MB842548.

Etymology: The species epithet refers to the county, where the type strain was collected, Cangyuan.

Type: China, Yunnan province, Cangyuan county, isolated as an endophyte from *Ageratina adenophora*, August 2016, Han-Bo Zhang. Holotype YMF 1.05001, Ex-type culture CGMCC3.18969.

Sexual morph not observed. Asexual morph on CMA. Conidiomata rudimentary, forming smaller aggregates of the mycelium with conidia droplets, dark olivaceous brown. Setae and conidiophores formed directly on hyphae. Setae scarce, cylindrical, straight or slightly flexuous, dark brown to black, 4–6-septate, base cylindrical to slightly inflated, tip acute to rounded, up to 102 μm. Conidiophores pale brown, aseptate, sometimes branched. Conidiogenous cells hyaline, smooth-walled, septate, subulate or cylindrical. Conidia hyaline, smooth-walled, cylindrical, slightly constricted at the middle, apex rounded, base obtuse, subtruncate, 15.5–20 × 4.5–5.5 μm, mean ± SD = 17.6 ± 1.3 × 5.1 ± 0.3 μm, l/w ratio = 3.5. Appressoria produced from mycelia, irregular, brown, branched, 12–16 × 6–11.5 μm.

Culture characteristics: Colonies on MEA flat forming a concentric ring with entire margin, occupied the whole plate in 7 days at 25 °C, surface grayish white, aerial mycelia medium hyaline to gray in the center, light gray to grayish white in the margin, partly covered with short felty whitish aerial mycelium, reverse same colors. Colonies on PDA flat forming a concentric ring with entire margin, occupied the whole plate in 7 days at 25 °C, aerial mycelia cotton-shaped, white to pale gray with circular margin, reverse same colors. Colonies on SNA flat with entire margin, occupied the whole plate in 7 days at 25 °C, surface covered with a radial, groove, thin, whitish aerial mycelium, reverse same colors. Colonies on CMA reaching 40–43 mm diameter in 7 days at 25 °C, aerial mycelium fluffy, flocculent, white, reverse same colors.

Additional specimens examined: China, Yunnan province, Cangyuan county, isolated as an endophyte from *Ageratina adenophora*, August 2016, Han-Bo Zhang, living cultures YMF 1.04998, YMF 1.05000.

Notes: Based on multi-locus phylogenetic analysis, three strains of *Colletotrichum cangyuanense* formed a sole clade in the gloeosporioides species complex, and the clade is close to *C. psidii* Curzi. Morphologically, *C. cangyuanense* shares similar morphological characteristics with other taxa in the gloeosporioides species complex, which have cylindrical conidia with rounded ends tapering slightly towards the base [66]. Nevertheless, *C. cangyuanense* is morphologically different from the phylogenetically nearest species *C. psidii* by having larger conidia, 15.5–20 × 4.5–5.5 vs. 12–15 × 3.5–4.5 μm [66].

**Figure 5:**
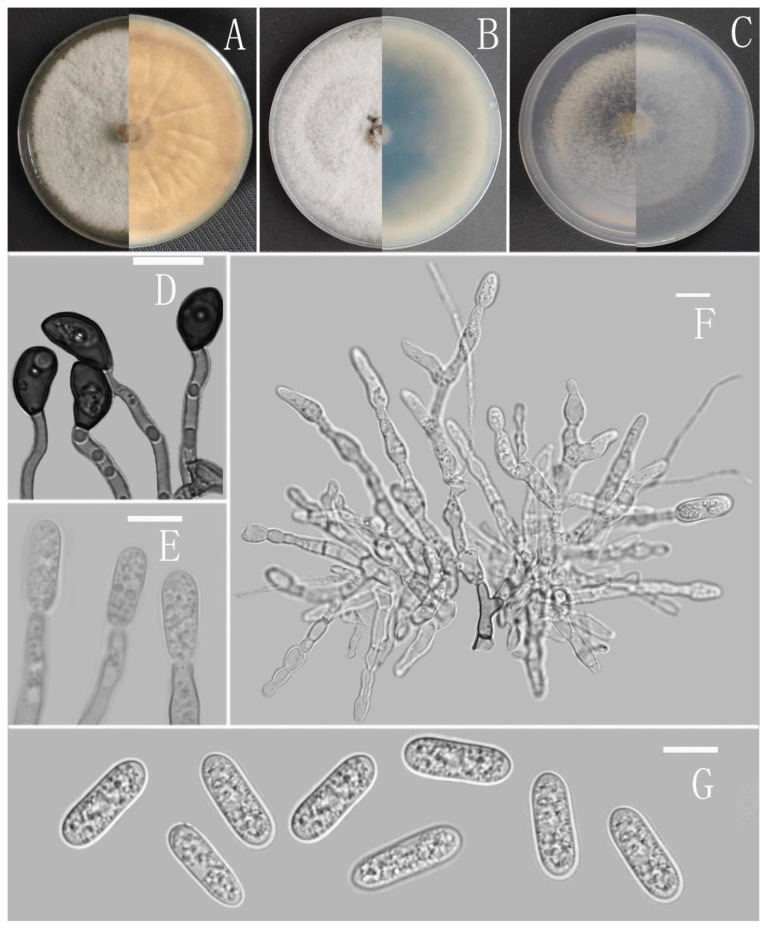
*Colletotrichum dimorphum* (YMF 1.07309). (**A**–**C**) Colonies on media above and below after 7 days at 25 °C ((**A**) MEA; (**B**) PDA; (**C**) SNA). (**D**) Appressoria. (**E**) Conidiophores. (**F**) Conidiophores and Conidia. (**G**) Conidia. Scale bars: (**D**–**G**) = 10 μm.

*Colletotrichum dimorphum* Z.F. Yu (Figure 5).

MycoBank Number: MB842549.

Etymology: The species epithet is based on the two forms of conidia.

Type: China, Guizhou province, Pingtang county, from disease spot of *Ageratina adenophora*, August 2018, Han-Bo Zhang. Holotype YMF 1.07309, Ex-type culture CGMCC3.16083.

Sexual morph not observed. Asexual morph on CMA. Conidiomata and setae not observed. Conidiophores directly formed on hyphae, hyaline, smooth-walled, unbranched. Conidiogenous cells hyaline, cylindrical, smooth-walled. Conidia hyaline, cylindrical to oblong, attenuate at the base, rounded at the apex, 10.5–19 × 4–6 μm, mean ± SD=14.6 ± 2 × 4.8 ± 0.7 μm, l/w ratio=3.1. Appressoria dark brown, aseptate, single, always elliptical in outline, the edge entire, 5.7–10.6 × 5–9 μm.

Culture characteristics: Colonies on MEA flat with entire margin, reaching 80 mm diameter in 7 days at 25 °C, surface grayish white, partly covered with fluffy, whitish aerial mycelium, light gray to grayish white in the margin, reverse medium grayish white to pale honey with brown pigmentation ring. Colonies on PDA flat with entire margin, occupied the whole plate in 7 days at 25 °C, aerial mycelia cotton-shaped, white to pale gray with circular margin, reverse iron grey to olivaceous grey. Colonies on SNA flat with entire margin, reaching 63–66 mm diameter in 7 days at 25 °C, aerial mycelia medium hyaline to grayish white in center, white to light gray in the margin, partly covered with short felty whitish aerial mycelium, reverse same colors. Colonies on CMA reaching 78 mm diameter in 7 days at 25 °C, aerial mycelium fluffy, flocculent, white, reverse same colors.

Additional specimen examined: China, Guizhou province, Pingtang county, from disease spot of *Ageratina adenophora*, August 2018, Han-Bo Zhang, living culture YMF1.07303.

Notes: Phylogenetically, *Colletotrichum dimorphum* fell into the gloeosporioides complex clade and near to the species *C. gloeosporioides*. Morphologically, *C. dimorphum* is distinguished from *C. gloeosporioides* by having slenderer conidia (l/w ratio: 3.1 vs. 2.6) and elliptical appressoria [67].

**Figure 6:**
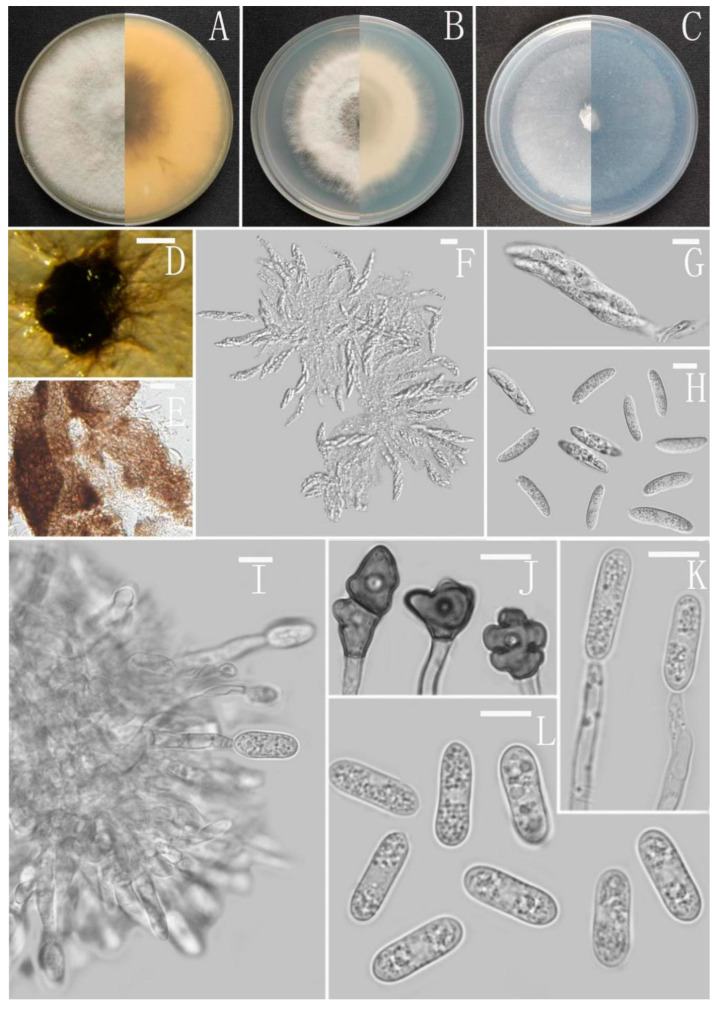
*Colletotrichum gracile* (YMF1.06939). (**A**–**C**) Colonies on media above and below after 7 days at 25 °C ((**A**) MEA; (**B**) PDA; (**C**) SNA). (**D**) Ascomata. (**E**) Outer surface of peridium. (**F**,**G**) Asci. (**H**) Ascospores. (**I**) Conidiophores and Conidia. (**J**) Appressoria. (**K**) Conidiophores. (**L**) Conidia. Scale bars: (**D**) = 100 μm, (**E**–**K**) = 10 μm.

*Colletotrichum gracile* Z.F. Yu (Figure 6).

MycoBank Number: MB842550.

Etymology: The species epithet refers to the slender and thin conidia.

Type: China, Guizhou province, Nayong county, from disease spot of *Ageratina adenophora*, August 2018, Han-Bo Zhang. Holotype YMF 1.06939, Ex-type culture CGMCC3.16075.

Sexual morph on CMA: Clusters of ascoshell aggregates of various sizes were formed on the colony in the later stage of the culture. Ascomata perithecia, solitary, superficial or immersed, non-stromatic, medium to dark brown, subglobose to pyriform, 358–320 × 257–267 µm. Peridium composed of medium brown flattened textura angularis with cells 3.2–4.9 µm diameter. Asci unitunicate, 8-spored, cylindrical, tapering to apex and base, smooth-walled, the 8 ascospores are arranged in a hemp rope shape, 55–60 × 12–15 µm. Ascospores uni- or biseriately arranged, hyaline smooth-walled, allantoid, curved most in the middle, with rounded ends, 15–18 × 2–3 μm, mean ± SD = 17 ± 1.1 × 3 ± 0.4 μm, l/w ratio = 5.7.

Asexual morph on CMA: Conidiomata and setae not observed. Conidiophores directly formed on hyphae, hyaline, smooth-walled, aseptate, unbranched. Conidiogenous cells hyaline, cylindrical to clavate, smooth-walled, collarette absent. Conidia cylindrical to clavate, aseptate, straight or slightly curved, both ends obtusely rounded or one end slightly acute, 13–18 × 4–6 μm, mean ± SD = 15 ± 1.1 × 5.2 ± 0.4 μm, l/w ratio = 2.9. Appressoria dark brown, aseptate, unbranched, single and little, irregular but often subglobose to ellipsoidal, margin sometimes lobed, 7–17.5 × 5–9 μm.

Culture characteristics: Colonies on MEA flat with entire margin, occupied the whole plate in 7 days at 25 °C, aerial mycelium fluffy, milky white, reverse brown to buff. Colonies on PDA reaching 72–76 mm diameter in 7 days at 25 °C, aerial mycelium dense, cottony, gray in the center and white in the margin, reverse same colors. Colonies on SNA flat with entire margin, reaching 70–72 mm diameter in 7 days at 25 °C, aerial mycelia medium hyaline to grayish white in center, white in the margin, partly covered with short whitish aerial mycelium, reverse same colors. Colonies growing on CMA with entire margin, reaching 64–66 mm diameter in 7 days at 25 °C, aerial mycelia medium gray to pale buff in center, light gray to grayish white in the margin, entirely covered with floccose to dense, producing pale gray conidiomata in 2 weeks. Reverse dark white to gray with white margin.

Additional specimen examined: China, Guizhou province, Nayong county, from disease spot of *Ageratina adenophora*, August 2018, Han-Bo Zhang, living culture YMF1.07329.

Notes: Based on multi-locus phylogenetic analysis, two strains of *Colletotrichum* gracile formed a solitary clade in the gloeosporioides complex clade, and phylogenetically near to the two new species *C. oblongisporum* and *C. nullisetosum*. However, *C. gracile* is easily distinguished from them by observing sexual morph.

**Figure 7:**
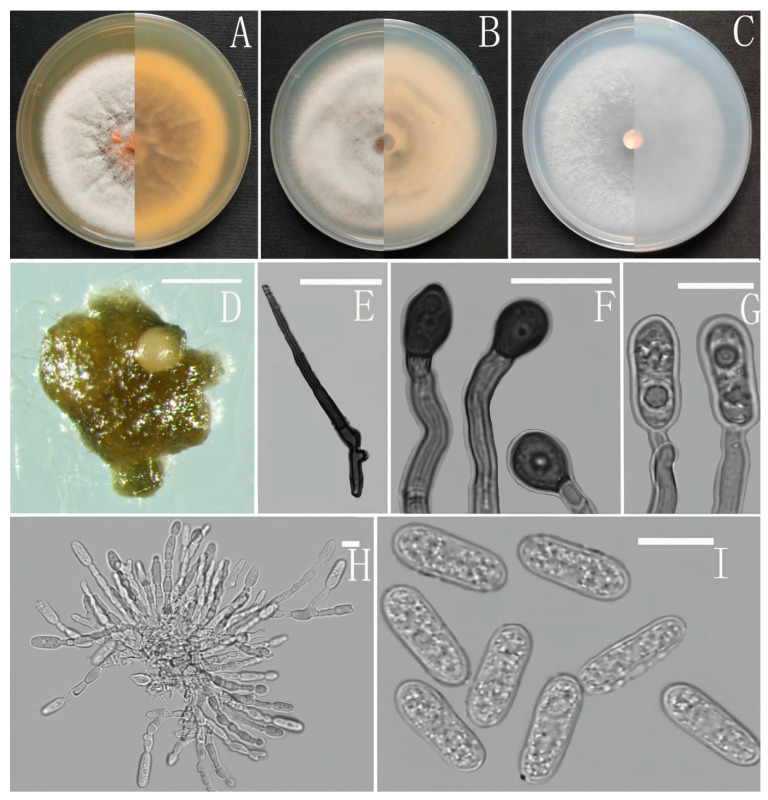
*Colletotrichum nanhuaense* (YMF1.04993). (**A**–**C**) Colonies on media above and below after 7 days at 25 °C ((**A**) MEA; (**B**) PDA; (**C**) SNA). (**D**) Conidiomata. (**E**) Seta. (**F**) Appressoria. (**G**) Conidiophores. (**H**) Conidiophores and Conidia. (**I**) Conidia. Scale bars: (**D**) = 100 μm, (**E**–**I**) = 10 μm.

*Colletotrichum nanhuaense* Z. F. Yu (Figure 7).

MycoBank Number: MB842551.

Etymology: The species epithet refers to the county, where the type strain was collected, Nanhua.

Type: China, Yunnan province, Nanhua county, from disease spot of *Ageratina adenophora*, August 2017, Han-Bo Zhang. Holotype YMF1.04993, Ex-type culture CGMCC3.18962.

Sexual morph not observed. Asexual morph on CMA. Conidiomata rudimentary, dark olivaceous brown. Setae and conidiophores formed directly on hyphae. Setae scarce, cylindrical, straight, dark brown to black, 3–5-septate, base bent with bulge, tip acute to rounded, 25 μm. Conidiophores hyaline to pale brown, aseptate, unbranched. Conidiogenous cells hyaline, cylindrical, smooth-walled, collarette absent. Conidia straight cylindrical, oblong to narrowly ovoid, obtuse at the base, rounded at the apex, contents granular, 10.5–16 × 4.5–6 μm, mean ± SD=14 ± 1.1 × 5.4 ± 0.4 μm, l/w ratio = 2.6. Appressoria dark brown, irregular, margin sometimes lobed, 8–14 × 5–8 μm.

Culture characteristics: Colonies on MEA flat with entire margin, reaching 68–72 mm diameter in 7 days at 25 °C, surface covered with radial, groove, felty, whitish aerial mycelium, Conidiomata acervular, with orange conidial masses, reverse iron gray to buff. Colonies on PDA flat with entire margin, reaching 76–79 mm diameter in 7 days at 25 °C, aerial mycelium dense, cottony, gray in the center and white in the margin, reverse carneose to grayish white. Colonies on SNA flat with entire margin, reaching 70–73 mm diameter in 7 days at 25 °C, aerial mycelia medium hyaline to grayish white in the center, white in the margin, partly covered with short whitish aerial mycelium, producing conidiomata with yellow conidial masses in 2 weeks, reverse same colors. Colonies growing on CMA with entire margin, 73–74 mm diameter in 7 days at 25 °C, aerial mycelia medium gray to pale buff in center, light gray to grayish white in the margin, entirely covered with floccose to dense, producing pale gray conidiomata in 2 weeks. Reverse dark white to grey with white margin.

Additional specimen examined: China, Yunnan province, Nanhua county, from disease spot of *Ageratina adenophora*, August 2017, Han-Bo Zhang, living culture YMF 1.04990.

Notes: Phylogenetically, *Colletotrichum nanhuaense* fell into the gloeosporioides complex clade and near to *C. gloeosporioides* and the new species *C. dimorphum*. Nevertheless, *C. nanhuaense* is morphologically different from them by having setae [67].

**Figure 8:**
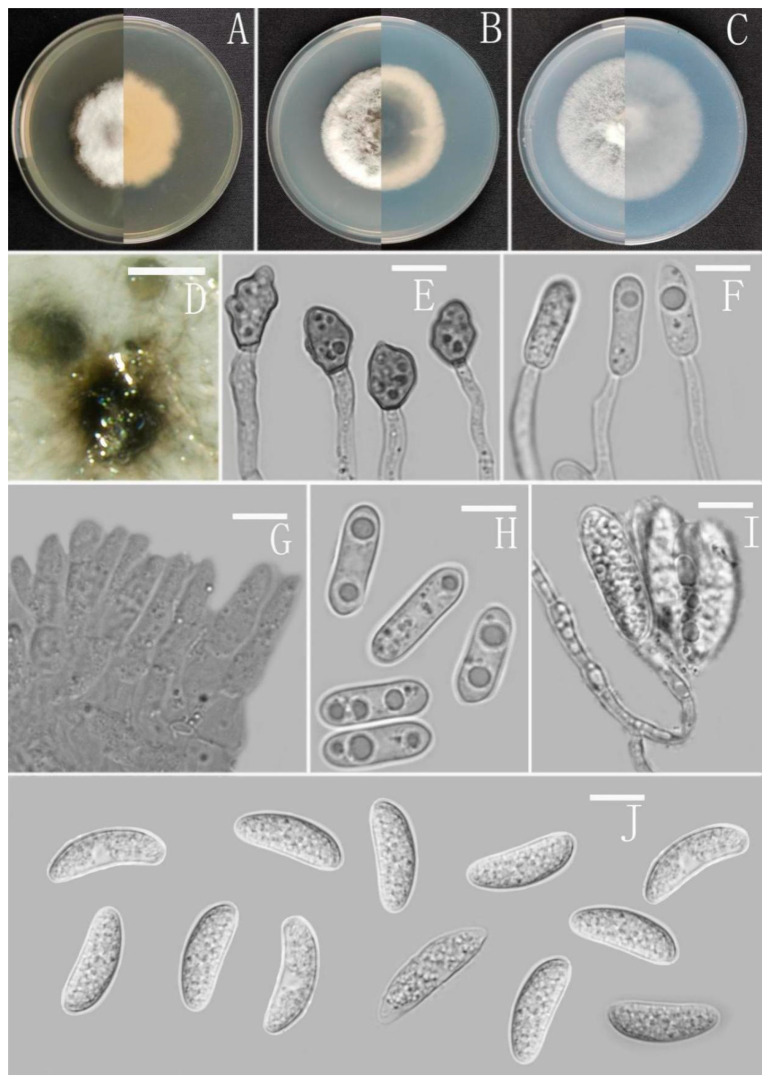
*Colletotrichum nullisetosum* (YMF1.06946). (**A**–**C**) Colonies on media above and below after 7 days at 25 °C ((**A**) MEA; (**B**) PDA; (**C**) SNA). (**D**) Conidiomata. (**E**) Appressoria. (**F**,**I**) Conidiophores. (**G**) Conidiophores and Conidia. (**H**,**J**) Conidia. Scale bars: (**D**) = 100 μm, (**E**–**J**) = 10 μm.

*Colletotrichum nullisetosum* Z.F. Yu (Figure 8).

MycoBank Number: MB842556.

Etymology: The species epithet is based on the absence of setae.

Type: China, Yunnan province, Ning’er county, from disease spot of mango, August 2017, Han-Bo Zhang. Holotype YMF1.06946, Ex-type culture CGMCC3.16080.

Sexual morph not observed. Asexual morph on CMA. Conidiomata rudimentary, dark olivaceous brown. Setae not observed. Conidiophores directly formed on hyphae, hyaline, smooth-walled, unbranched. Conidiogenous cells hyaline, cylindrical to clavate, smooth-walled, collarette absent. Conidia unicellular, hyaline, dimorphic; (i) straight cylindrical, oblong to narrowly ovoid, obtuse at the base, rounded at the apex, contents 1–2-granular, 13.5–19.5 × 4–6.5 μm, mean ± SD = 15.5 ± 1.3 × 5 ± 0.5 μm, l/w ratio = 3.1. (ii) sickle, aseptate, blunt at both ends, curved to one side, contents 1-granular, 14.8–19.3 × 5–6.5, mean ± SD = 17.3 ± 1.1 × 5.8 ± 0.3 μm, l/w ratio = 3.0. Appressoria dark brown, sometimes branched, always little and oval in outline, contents granular, 9–15 × 5–8 μm.

Culture characteristics: Colonies on MEA reaching 44–45 mm diameter in 7 days at 25 °C, aerial mycelia cotton-shaped, grayish white to white, turning iron gray in the later stage, reverse same colors. Colonies on PDA reaching 49–51 mm diameter in 4 days at 25 °C, aerial mycelia cotton-shaped, white to pale gray with circular margin, reverse iron gray to olivaceous gray. Conidial mass whitish to pale yellow. Colonies on SNA reaching 58–60 mm diameter in 7 days at 25 °C, aerial mycelium fluffy, flocculent, white, producing black conidiomata in 2 weeks, reverse same colors. Colonies on CMA reaching 57–60 mm diameter in 7 days at 25 °C, aerial mycelium fluffy, flocculent, white, reverse same colors.

Additional specimen examined: China, Yunnan province, Ning’er county, from disease spot of the common fruit mango, August 2017, Han-Bo Zhang, living culture YMF 1.07328.

Notes: *Colletotrichum nullisetosum* is phylogenetically close to the new species *C. oblongisporum*, but it can be distinguished from *C. oblongisporum* by having dimorphic conidia.

**Figure 9:**
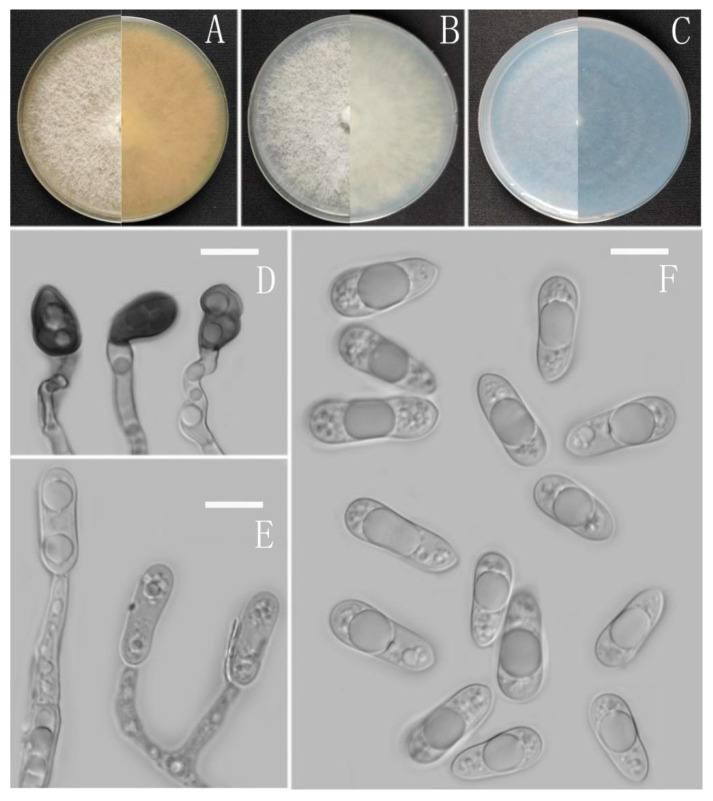
*Colletotrichum oblongisporum* (YMF 1.06938). (**A**–**C**) Colonies on media above and below after 7 days at 25 °C ((**A**) MEA; (**B**) PDA; (**C**) SNA). (**D**) Appressoria. (**E**) Conidiophores. (**F**) Conidia. Scale bars: (**D**–**F**) = 10 μm.

*Colletotrichum oblongisporum* Z.F. Yu (Figure 9).

MycoBank Number: MB842557.

Etymology: The species epithet refers to oblong shape of conidia.

Type: China, Yunnan province, Kunming city, Xishan Park, isolated as an endophyte from *Ageratina adenophora*, September 2017, Han-Bo Zhang. Holotype YMF1.06938, Ex-type culture CGMCC3.16074.

Sexual morph not observed. Asexual morph on CMA. Conidiomata and setae not observed. Conidiophores directly formed on hyphae, hyaline, smooth-walled, septate, occasionally branched. Conidiogenous cells hyaline, cylindrical, collarette not visible. Conidia oblong, unicellular, subtruncate at the base, biguttulate near the ends, hyaline, smooth-walled, 13–18.5 × 4–5.5 μm, mean ± SD = 15.3 ± 1.4 × 4.7 ± 0.4 μm, l/w ratio = 3.3. Appressoria dark brown, subglobose, cuneiform to irregular, sometimes with lobate margin, 8–15 × 4.5–7.5 μm.

Culture characteristics: Colonies on MEA flat with entire margin, occupied the whole plate in 7 days at 25 °C, aerial mycelium fluffy, flocculent, white on the edges, reverse white to light yellow. Colonies on PDA flat with entire margin, occupied the whole plate in 7 days at 25 °C, aerial mycelium dense, cottony, in the later stage of culture, formed a four concentric ring pattern, from the center to the edge, pale gray to dark gray. Colonies on SNA flat with entire margin, occupied the whole plate in 7 days at 25 °C, aerial mycelium white, formed in the shape of four concentric ring pattern, reverse same colors. Colonies on CMA flat with entire margin, occupied the whole plate in 7 days at 25 °C, aerial mycelium fluffy, flocculent, white, reverse same colors.

Additional specimen examined: China, Yunnan province, Kunming city, Xishan Park, isolated as an endophyte from *Ageratina adenophora*, September 2017, Han-Bo Zhang, living culture YMF 1.07326.

Notes: Based on multi-locus phylogenetic analysis, two strains of *Colletotrichum oblongisporum* formed a solitary clade in the gloeosporioides complex clade, and phylogenetically near to the two new species *C. oblongisporum* and *C. nullisetosum*, but it can be easily distinguished from them in morphology. For instance, *C. gracile* differs from *C. oblongisporum* in the shape of conidia and appressoria. *C. gracile* has cylindrical to clavate conidia and subglobose to ellipsoidal appressoria, while *C. oblongisporum* has oblong conidia and subglobose and cuneiform to irregular appressoria.

**Figure 10:**
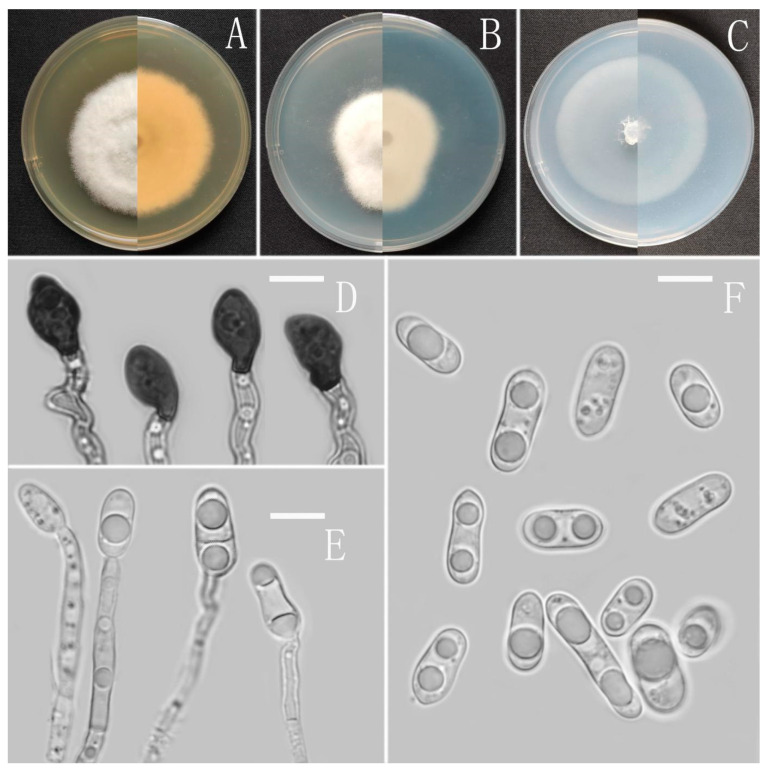
*Colletotrichum parvisporum* (YMF1.06942). (**A**–**C**) Colonies on media above and below after 7 days at 25 °C ((**A**) MEA; (**B**) PDA; (**C**) SNA). (**D**) Appressoria. (**E**) Conidiophores. (**F**) Conidia. Scale bars: (**D**–**F**) = 10 μm.

*Colletotrichum parvisporum* Z.F. Yu (Figure 10).

MycoBank Number: MB842558.

Etymology: The species epithet is based on the relatively smaller conidia.

Type: China, Guangxi province, Debao county, from disease spot of *Ageratina adenophora*, August 2018, Han-Bo Zhang. Holotype YMF 1.06942, Ex-type culture CGMCC3.16078.

Sexual morph not observed. Asexual morph on CMA. Conidiomata and setae not observed. Conidiophores formed directly from mycelia, hyaline, sometimes branched. Conidiogenous cells hyaline, smooth-walled, cylindrical or oval, collarette not visible. Conidia oval or cylindrical, both with broadly rounded ends, straight or sometimes slightly bent, sometimes contracted in the middle, the contents are circular, 10–15 × 3.5–7.5 μm, mean ± SD = 12.5 ± 1.3 × 5.8 ± 0.9 μm, l/w ratio = 2.2. Appressoria produced from mycelia, clavate or irregular outline, aseptate, brown, 7.5–17 × 4.5–10 μm, mean ± SD = 11.7 ± 2.1 × 7.2 ± 1.6 μm.

Culture characteristics: Colonies on MEA reaching 55–58 mm diameter in 7 days at 25 °C, aerial mycelia cotton-shaped, grayish white to white, turning to iron gray in the later stage, reverse same colors. Colonies on PDA reaching 40–49 mm diameter in 5 days at 25 °C, felted aerial mycelia with grayish white appearance, reverse grayish white. Colonies on SNA flat with entire margin, reaching 65–69 mm diameter in 7 days at 25 °C, aerial mycelia medium hyaline to grayish white in center, white in the margin, partly covered with short whitish aerial mycelium, reverse same colors. Colonies on CMA flat with entire margin, reaching 50–54 mm diameter in 7 days at 25 °C, aerial mycelia hyaline to greyish white in center, white in the margin, partly covered with short whitish aerial mycelium, reverse same colors.

Notes: Phylogenetically, *Colletotrichum parvisporum* clustered with *C. pandanicola* Tibpromma and K.D. Hyde in the gloeosporioides complex clade. *C. pandanicola* was introduced by Tibpromma et al. in 2018; it was recorded as an endophyte isolated from the leaves of *Pandanus* sp. in Thailand and sexual morph was not reported [68]. Morphologically, *C. pandanicola* is easily distinguished from the new species *C. parvisporum* by having cylindrical conidia and lacking appressoria [68].

**Figure 11:**
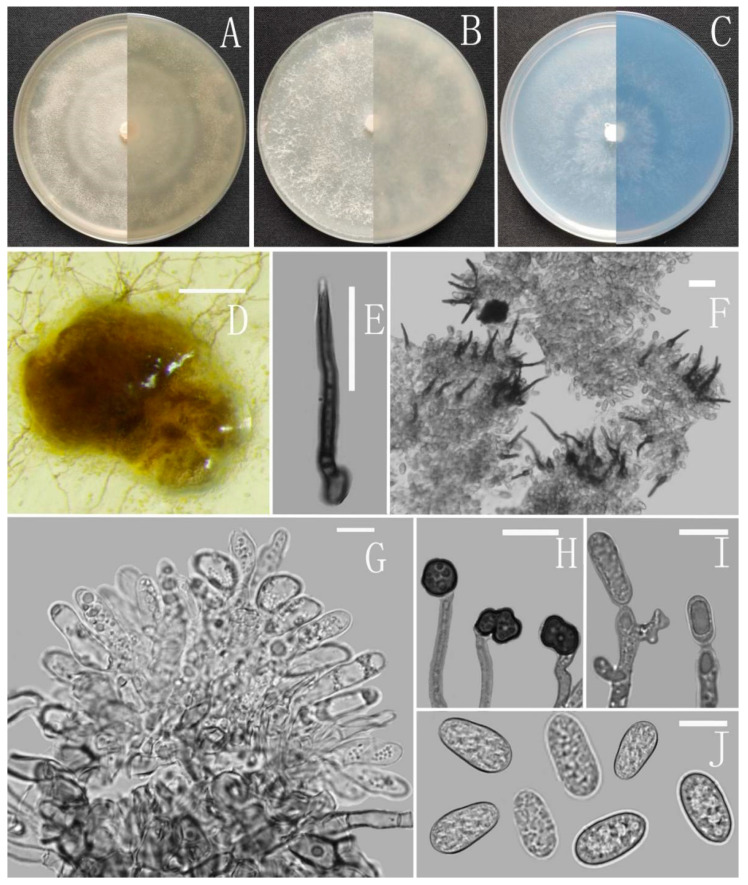
*Colletotrichum robustum* (YMF1.06941). (**A**–**C**) Colonies on media above and below after 7 days at 25 °C ((**A**) MEA; (**B**) PDA; (**C**) SNA). (**D**) Conidiomata. (**E**,**F**) Setae. (**G**) Conidiophores and Conidia. (**H**) Appressoria. (**I**) Conidiophores. (**J**) Conidia. Scale bars: (**D**) = 100 μm, (**F**) = 20 μm, (**E**,**G**–**J**) = 10 μm.

*Colletotrichum robustum* Z. F. Yu (Figure 11).

MycoBank Number: MB842559.

Etymology: The species epithet is based on the conidia, meaning robust, strong and vigorous.

Type: China, Guizhou province, Dushan county, on disease spot of *Ageratina adenophora*, August 2018, Han-Bo Zhang. Holotype YMF 1.06941, Ex-type culture CGMCC3.16077.

Sexual morph not observed. Asexual morph on CMA. Conidiomata rudimentary, pale brown. Setae and conidiophores formed directly on hyphae. Setae abundant, cylindrical, straight or slightly flexuous, dark brown to black, 3–5-septate, base cylindrical to slightly inflated, tip acute to rounded, 11.8–15.1 μm long. Conidiophores abundant, hyaline, branched. Conidiogenous cells hyaline, cylindrical to clavate, collarette not visible. Conidia smooth-walled, hyaline, broad cylindrical, rounded at the ends, sometimes slightly constricted in the middle, the circular or elliptical guttulate. 12.5–16.0 × 5.5–7.5 μm, mean ± SD = 14.0 ± 0.8 × 6.5 ± 0.5 μm, l/w ratio = 2.2. Appressoria circular or irregular outline, brown, branched, 6.0–8.0 × 5.0–7.0 μm.

Culture characteristics: Colonies on MEA flat forming a concentric ring with entire margin, occupied the whole plate in 7 days at 25 °C, surface grayish white, aerial mycelia medium hyaline to gray in center, light gray to grayish white in the margin, partly covered with short felty whitish aerial mycelium, reverse same colors. Colonies on PDA flat with entire margin, occupied the whole plate in 7 days at 25 °C, aerial mycelium fluffy, flocculent, white in edges, reverse white to light yellow, aerial mycelium sparse, white to pale gray, with copious yellow conidial masses in the later stage of culture, reverse pale luteous to sienna with circinate black colored pigmentation. Colonies on SNA flat is concentric ring with entire margin, occupied the whole plate in 7 days at 25 °C, aerial mycelia medium white to hyaline in center, partly covered with short felty whitish aerial mycelium, reverse same colors. Colonies on CMA flat with entire margin, reaching 68 mm diameter in 7 days at 25 °C, aerial mycelia medium hyaline to grayish white in center, white in the margin, partly covered with a short, whitish aerial mycelium, reverse same colors.

Notes: In this phylogenetic tree, *Colletotrichum robustum* fell into the dracaenophium complex clade, and clustered with *C. excelsum-altitudinum* G. Tao, Zuo Y. Liu and L. Cai and *C. tongrenense* S.X. Zhou, J.C. Kang and K.D. Hyde. However, *C. robustum* can be distinguished from them in morphology. *C. excelsum-altitudinum* was isolated as an endophyte from *Bletilla ochracea* in Guizhou [42]. It has larger conidia and appressoria than *C. robustum* but has a slower growth rate on PDA. *C. tongrenense* was also isolated as an endophyte from *Nothapodytes pittosporoides* in Guizhou [69]. *C. tongrenense* is obviously different in having shorter conidia, 11–14 × 5–7 μm, and lacking appressoria.

**Figure 12:**
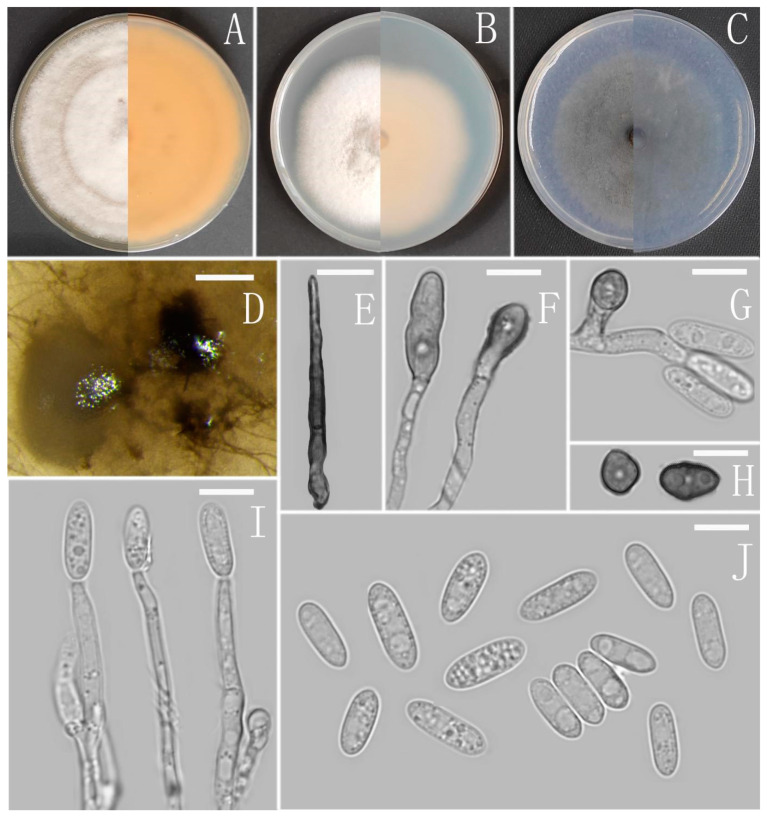
*Colletotrichum simulanticitri* (YMF1.07302). (**A**–**C**) Colonies on media above and below after 7 days at 25 °C ((**A**) MEA; (**B**) PDA; (**C**) SNA). (**D**) Conidiomata. (**E**) Setae. (**F**,**H**) Appressoria. (**G**) Appressoria and Conidia. (**I**) Conidiophores. (**J**) Conidia. Scale bars: (**D**) = 100 μm, (**E**–**J**) = 10 μm.

*Colletotrichum simulanticitri* Z. F. Yu (Figure 12).

MycoBank Number: MB842560.

Etymology: The species epithet is based on the similarity to *Colletotrichum citri*.

Type: China, Yunnan province, Dali county, from disease spot of *Betula* sp., August 2016, Han-Bo Zhang. Holotype YMF 1.07302, Ex-type culture CGMCC 3.16082.

Sexual morph not observed. Asexual morph on CMA. Conidiomata rudimentary, stromatic, subpulvinate, dark brown. Setae cylindrical to subulate, obtuse at the apex, 1–2-septate, smooth-walled, dark brown to black, 33–44 μm. Conidiophores abundant, hyaline, smooth-walled, septate, branched. Conidiogenous cells hyaline, cylindrical. Conidia hyaline, cylindrical to oblong, attenuated at the base, rounded at the apex, unicellular, guttulate, 10–13.5 × 4–5 μm, mean ± SD = 12 ± 1 × 4.3 ± 0.3 μm, l/w ratio = 2.8. Appressoria dark brown, septate, clavate or oval, smooth margin, 6–15.5 × 4–5.5 μm.

Culture characteristics: Colonies on MEA flat forming a concentric ring with entire margin, occupied the whole plate in 7 days at 25 °C, white to grayish white in the margin, partly covered with short felty whitish aerial mycelium, reverse white to buff. Colonies on PDA reaching 60–63 mm diameter in 7 d at 25 °C, felted aerial mycelia with grayish white appearance, reverse grayish white, aerial mycelia turning dark gray and later becoming cotton-like lately. Colonies on SNA flat with entire margin, reaching 59–61 mm diameter in 7 days at 25 °C, aerial mycelia medium iron gray in center, light gray in the margin, partly covered with short felty gray aerial mycelium, reverse same colors. Colonies on CMA flat with entire margin, reaching 57–61 mm diameter in 7 days at 25 °C, aerial mycelia dark brown, conidiomata scarced, dark, reverse same colors.

Additional specimens examined: China, Yunnan province, Dali county, from disease spot of Betula sp., August 2016, Han-Bo Zhang, living cultures YMF 1.07312, YMF 1.07308.

Notes: Based on multi-locus phylogenetic analysis, three strains of *Colletotrichum simulanticitri* formed a solitary clade in the gloeosporioides complex clade and near to *C. nymphaeae* (Pass.) Aa and *C. citri* F. Huang, L. Cai, K.D. Hyde and Hong Y. Li. Morphologically, *C. simulanticitri* can be distinguished from *C. nymphaeae* by having setae and and shorter conidia (10–13.5 μm vs. 14–18.5 μm) [70]. *C. simulanticitri* is distinguished from *C. citri* by the conidia shape and size. The shape conidia of *C. citri* is fusiform and acute to near-rounded at two ends, while *C. simulanticitri* is cylindrical to oblong and attenuate at the base and rounded at the apex [38]. Moreover, the conidia of *C. simulanticitri* are wider than *C. citri*, 4–5 μm vs. 3.3–4.4 μm.

**Figure 13:**
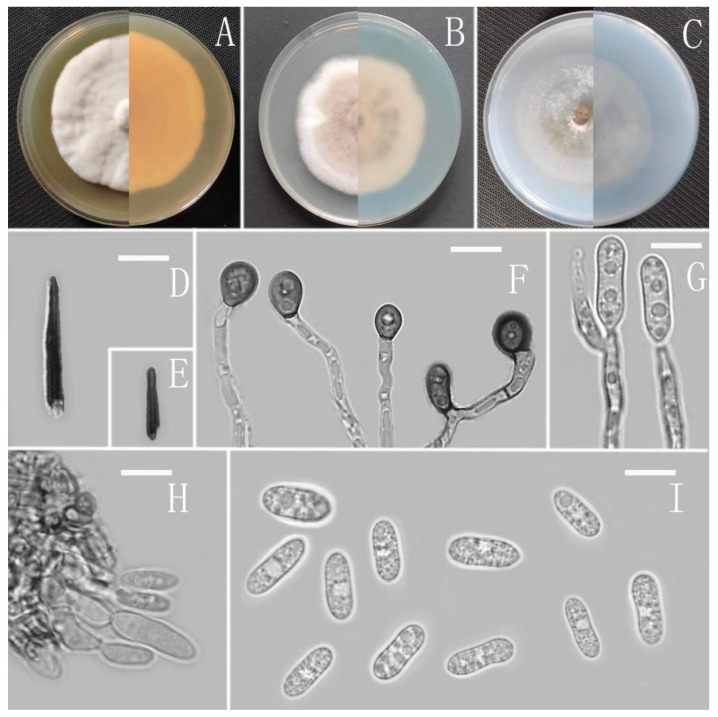
*Colletotrichum speciosum* (YMF1.07301). (**A**–**C**) Colonies on media above and below after 7 days at 25 °C ((**A**) MEA; (**B**) PDA; (**C**) SNA). (**D**,**E**) Setae. (**F**) Appressoria. (**G**) Conidiophores. (**H**) Conidiophores and Conidia. (**I**) Conidia. Scale bars: (**D**–**I**) = 10 μm.

*Colletotrichum speciosum* Z.F. Yu (Figure 13).

MycoBank Number: MB842562.

Etymology: The species epithet refers to the showy and splendid culture characteristics. Type: China, Yunnan province, Simao county, isolated as an endophyte from *Ageratina adenophora*, August 2016, Han-Bo Zhang. Holotype YMF 1.07301, Ex-type culture CGMCC3.16081.

Sexual morph not observed. Asexual morph on CMA. Conidiomata not observed. Setae scarce, cylindrical, straight, dark brown to black, 1–2-septate, obtuse or with a bulge at the apex, tip acute to rounded, 12–21 μm. Conidiophores formed directly on aerial mycelium, hyaline, 0–1-septate, sometimes branched. Conidiogenous cells hyaline, thin cylindrical to clavate, collarette inconspicuous. Conidia hyaline, unicellular, smooth-walled, cylindrical, slightly constricted at the middle, attenuated at the base, rounded at the apex, lumen granular, 10.5–14.5 × 4–5.5 μm, mean ± SD = 12.5 ± 1.0 × 4.8 ± 0.4 μm, l/w ratio = 2.6. Appressoria dark brown, broadly oboval to obovoid or subglobose, the margin smooth, 7–9.5 × 4.5–7.5 μm.

Culture characteristics: Colonies on MEA flat forming concentric ring with radial groove, reaching 65–67 mm diameter in 7 days at 25 °C, covered with short, felty, whitish aerial mycelium, reverse white to buff. Colonies on PDA reaching 59–61 mm diameter in 7 days at 25 °C, covered with felty aerial mycelium, whitish to pale gray with circular margin, reverse olivaceous gray to pale gray. Colonies on SNA flat with entire margin, reaching 58–60 mm diameter in 7 days at 25 °C, aerial mycelia medium grayish white in the center, light gray to hyaline in the margin, reverse pale gray to hyaline. Colonies on CMA flat with entire margin, reaching 59 mm diameter in 7 days at 25 °C, covered with thin whitish aerial mycelium, producing dark conidiomata in 2 weeks, reverse same colors.

Notes: The phylogenetic analyses based on multi-locus revealed that *Colletotrichum speciosum* belongs to the acutatum species complex and is closely related to *C. chrysanthemi* (Hori) Sawada and *C. carthami* (Fukui) S. Uematsu, Kageyama, Moriwaki & Toy. Sato. However, *C. chrysanthemi* is morphologically different from *C. speciosum* by its very short acute-ended conidia [70]. Furthermore, *C. carthami* also differs from *C. speciosum* by having fusiform to oblong and narrower (2.6–4.6 μm) conidia [71].

**Figure 14:**
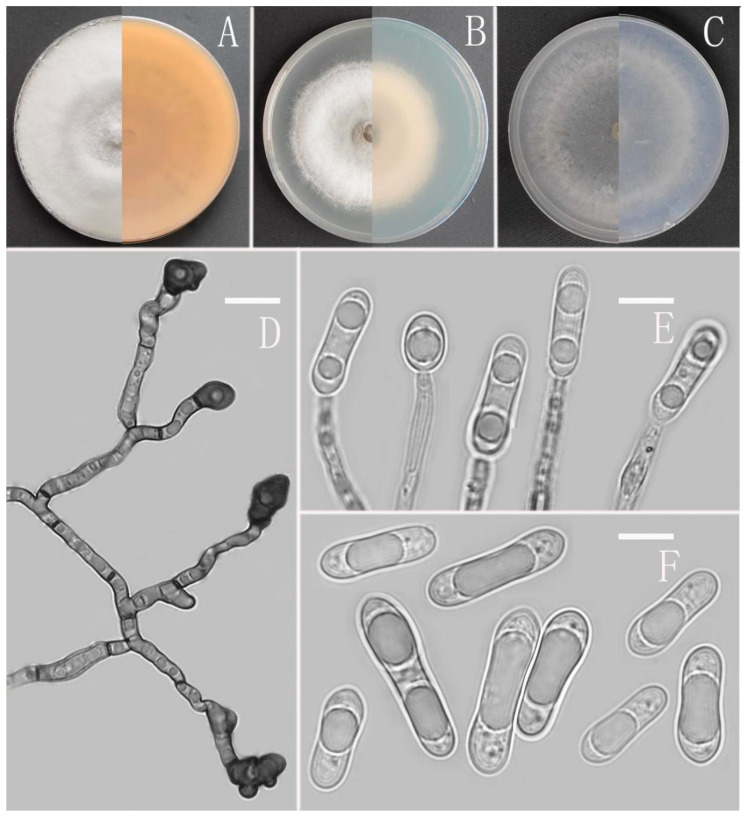
*Colletotrichum subhenanense* (YMF1.06865). (**A**–**C**) Colonies on media above and below after 7 d at 25 °C ((**A**) MEA; (**B**) PDA; (**C**) SNA). (**D**) Appressoria. (**E**) Conidiophores. (**F**) Conidia. Scale bars: (**D**–**F**) = 10 μm.

*Colletotrichum subhenanense* Z.F. Yu (Figure 14).

MycoBank Number: MB842563.

Etymology: The species epithet is based on the species *Colletotrichum henanense*, which is phylogenetically closely related.

Type: China, Yunnan province, Cangyuan county, from disease spot of *Ageratina adenophora*, August 2016, Han-Bo Zhang. Holotype YMF 1.06865, Ex-type culture CGMCC3.16073.

Sexual morph not observed. Asexual morph on CMA. Conidiomata and setae not observed. Conidiophores directly formed on hyphae, hyaline, smooth-walled, septate, sometimes branched. Conidiogenous cells hyaline, cylindrical, smooth-walled, collarette absent. Conidia plump cylindrical, oblong to narrowly ovoid, unicellular, hyaline, guttulate, straight or slightly curved, obtuse at the base, rounded at the apex, 10–20 × 4–6.5 μm, mean ± SD = 14.3 ± 1.9 × 5 ± 0.6 μm, l/w ratio = 2.9. Appressoria dark brown, septate, single, irregular but often subglobose in outline, margin smooth, 7–13 × 6–10 μm.

Culture characteristics: Colonies on MEA flat with entire margin, occupied the whole plate in 7 days at 25 °C, aerial mycelium fluffy, milky white, reverse pale gray to buff. Colonies on PDA reaching 59–62 mm diameter in 7 days at 25 °C, aerial mycelium dense, cottony, gray in the center and white in the margin, turning dark later, reverse olivaceous to grayish white. Colonies on SNA flat with entire margin, reaching 85–88 mm diameter in 7 days at 25 °C, aerial mycelia medium hyaline to grayish white in the center, white in the margin, partly covered with short, whitish aerial mycelium, reverse same colors. Colonies on CMA reaching 75–78 mm diameter in 7 days at 25 °C, aerial mycelium fluffy, flocculent, white, turning dark later, reverse same colors.

Additional specimen examined: China, Yunnan province, Cangyuan county, from disease spot of *Ageratina adenophora*, August 2016, Han-Bo Zhang, living culture YMF 1.07324.

Notes: Based on multi-locus phylogenetic analysis, two strains of *Colletotrichum subhenanense* formed a solitary clade in the gloeosporioides complex clade, and near to *C. henanense* F. Liu and L. Cai, *C. yulongense* C.L. Hou and X.T. Liu, and *C. hederiicola* Jayaward. Camporesi and K.D. Hyde. Morphologically, *C. subhenanense* is most similar to *C. yulongense* in culture characteristics on PAD and SNA, with cylindrical conidia with obtuse to slightly rounded ends, but *C. yulongense* is obviously distinguished by having septate setae and narrower conidia (4–5 μm) [72].

**Figure 15:**
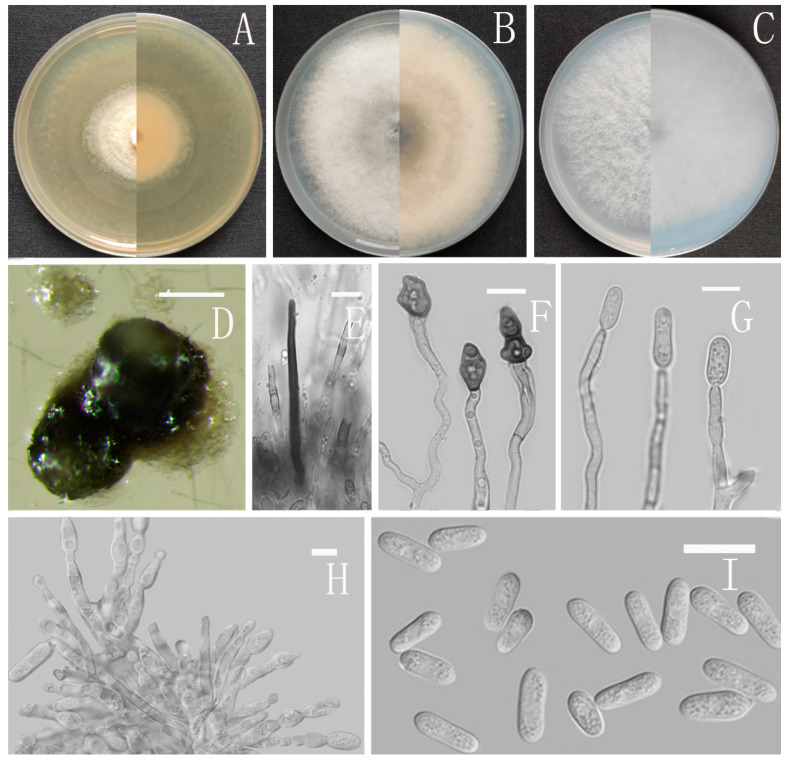
*Colletotrichum yunajiangense* (YMF1.04996). (**A**–**C**) Colonies on media above and below after 7 days at 25 °C ((**A**) MEA; (**B**) PDA; (**C**) SNA). (**D**) Conidiomata. (**E**) Setae. (**F**) Appressoria. (**G**) Conidiophores. (**H**) Conidiophores and Conidia. (**I**) Conidia. Scale bars: (**D**) = 100 μm, (**E**–**I**) = 10 μm.

*Colletotrichum yuanjiangense* Z.F. Yu (Figure 15).

MycoBank Number: MB842564.

Etymology: The species epithet is derived from the county, where the type strain was collected, Yuanjiang.

Type: China, Yunnan province, Yuanjiang county, on disease spot of *Ageratina adenophora*, August 2017, Han-Bo Zhang. Holotype YMF 1.04996, Ex-type culture CGMCC3.18964.

Sexual morph not observed. Asexual morph on CMA. Conidiomata rudimentary, dark olivaceous brown. Setae and conidiophores formed directly on hyphae. Setae scarce, cylindrical, straight or slightly flexuous, dark brown to black, 4–6-septate, base cylindrical to slightly inflated, tip acute to rounded, 62 μm. Conidiophores directly formed on hyphae, hyaline, smooth-walled, aseptate, unbranched. Conidiogenous cells hyaline, cylindrical to clavate, smooth-walled, collarette absent. Conidia hyaline, smooth-walled, cylindrical, obtuse at both ends or slightly acute at one end, 10–14 × 4–6 μm, mean ± SD=12 ± 0.9 × 5.2 ± 0.5 μm, l/w ratio = 2.3. Appressoria dark brown, aseptate, single, subglobose or elliptical, the edge entire, sometimes slightly lobed, 7–13.5 × 6–10 μm.

Culture characteristics: Colonies on MEA flat with entire margin, reaching 38–42 mm diameter in 7 days at 25 °C, aerial mycelia medium buff to white, hyaline in the margin, partly covered with a short, whitish aerial mycelium, reverse buff to pale honey. Colonies on PDA reaching 80 mm diameter in 7 days at 25 °C, aerial mycelium cottony, pale gray in the center, grayish white at the edge, reverse olivaceous to grayish white. Colonies on SNA flat with entire margin, reaching 68–73 mm diameter in 7 days at 25 °C, aerial mycelia medium hyaline to white in center, white in the margin, partly covered with short whitish aerial mycelium, reverse same colors. Colonies growing on CMA with entire margin, 72–74 mm diameter in 7 days at 25 °C, aerial mycelia medium gray to pale buff in center, light gray to grayish white in the margin, entirely covered with floccose to dense, producing pale gray conidiomata in 2 weeks. Reverse same colors.

Additional specimen examined: China, Yunnan province, Yuanjiang county, from disease spot of *Ageratina adenophora*, August 2017, Han-Bo Zhang, living culture YMF 1.04997.

Notes: In this phylogenetic tree, *Colletotrichum yuanjiangense* fell into the gloeosporioides complex clade, and near to the two new species, *C. nanhuaense* and *C. dimorphum*, and *C. gloeosporioides*. Morphologically, *C. yuanjiangense* is most similar to *C. nanhuaense* in having septate setae and cylindrical conidia, but it can be distinguished by having subglobose or elliptical appressoria with an irregular edge.

## 4. Discussion

Although plant invasion is common and intensively studied [73,74], fungi associated with the host have received relatively less attention. A few cases have indicated that diverse endophytes were found in the leaves of invasive plants, so these fungi were co-introduced as well as local infection [16]. Regardless of the case, fungus identity is less known. Invasive plants invade a new range and commonly establish novel associations with local microbes [75,76]. Thus, invasive plants are good sources for novel microbial species. In this study, it is surprising that the diversity of *Colletotrichum* species was so abundant in *Ageratina* *adenophora*. We revealed 26 species associated with seven species complexes, including 14 new species *C. adenophorae*, *C. analogum*, *C. cangyuanense*, *C. dimorphum*, *C.* *gracile*, *C. nanhuaense*, *C. nullisetosum*, *C. oblongisporum*, *C. parvisporum*, *C. robustum*, *C. simulanticitri*, *C. speciosum*, *C. subhenanense*, *C. yunajiangense* and 12 known species *C. boninense*, *C. citri*, *C. cosmi*, *C. fioriniae*, *C. fructicola*, *C. gigasporum*, *C. godetiae*, *C. guajavae*, *C. karstii*, *C. magnum*, *C. nymphaeae*, *C. plurivorum*.

Although *Colletotrichum* is a common endophyte in most plants, its association with specific fungal taxa or its high relative abundance suggests it has a major effect on plant communities, given that *Colletotrichum* consistently occurs abundantly in the endophyte communities of *A. adenophora*. We hypothesized a pathogenic role in *A. adenophora*. The detection of many *Colletotrichum* spp., including the 14 novel species, in this study indicated the potential of *Colletotrichum* spread with *A. adenophora* invasion. The pathogenicity of these *Colletotrichum* strains (not shown here) indicated the necessity of investigating fungal endophytes in *A. adenphora*, as well as other invasive plants.

Recent evidence has indicated that the diversity of pathogens on highly abundant introduced hosts has been positively correlated over time with the geographical range of the introduced species and the diversity of invaded habitats [77]. Invasive species *Microstegium vimineum* (Trin.) *A. Camus* has been proven to be infected by *Bipolaris* spp. Shoemaker in the eastern USA [78]. Day et al. [26] isolated fungi from the roots of invasive *Vincetoxicum rossicum* (Kleopow) Barbar. (Apocynaceae) plants collected across Ontario, Canada, and indicated that some of them can reduce the total biomass of a native species *Solidago Canadensis* L., which may be important for mediating plant community structure and the persistence of invasive plants. Several studies have also found accompanying changes in populations of specific soil pathogens and their impacts on invasive and noninvasive species [79,80]. Recently, fungi isolated from the invasive plant *Vincetoxicum rossicum* have been proven to have differential growth effects between non-native invasive and native plants, which may be important for mediating plant community structure and the persistence of invasive plants [26]. Accumulations of pathogens are common in several detected invasive plants, including above-ground, for example, leaves [78], and belowground, e.g., soil oomycete pathogens in freshwater wetland soils invaded by non-native *Phragmites australis* (Cav.) Trin. ex Steud. (European common reed) [80]. We found that rhizosphere soils of *Chromolaena odorata* L., one of the world’s most destructive tropical invasive weeds, accumulate high concentrations of the generalist soil-borne fungi, *Fusarium* Link (tentatively identified as *F. semitectum*), thus creating a negative feedback for native plant species [79]. If *Colletotrichum* is pathogenic on *A. adenophora*, an abundance of this species may alleviate the spread of *A. adenophora*. If it is pathogenic on native plants, including fruits, the risk is great and should be emphasized in future.

Previously, association with the fungal endophyte *Neotyphodium coenophialum* (Morgan-Jones and W. Gams) Glenn et al. increased the ability of *Lolium arundinaceum* (Schreb.) S.J. Darbyshire to invade communities with greater species diversity. In the absence of the endophyte, the initial diversity of the community significantly reduced the establishment of *L. arundinaceum*. However, establishment was independent of initial diversity in the presence of the endophyte [81]. We tested the impact of the fungal endophyte *Alternaria alternata* (Fr.) Keissl. (phylotype CID 120) on the allelopathic effect of the invasive forb *Centaurea stoebe* when in competition with the North American native bunchgrass *Koeleria macrantha* (Ledeb.) Schultes in a greenhouse competition experiment. The allelopathic effect on *K. macrantha* of *C. stoebe* when infected with the fungal endophyte was more than twice that of endophyte-free *C. stoebe*. Fungal endophytes may increase the competitive ability of their hosts [23]. Two phylotypes of *Alternaria* endophytes influenced the growth, competitive effects, and competitive responses of the exotic invasive forb *C. stoebe*. The competitive effects of *C. stoebe* on grass species native to North America were enhanced by both fungal endophytes [24]. Arnold et al. [40] indicated that *Colletotrichum*-enhanced cacao trees resist pathogen attack. In this case, the role of the foliar fungus *Colletotrichum* sp. may be a weak latent foliar pathogen of *A. adenophora* and can enhance the pathogenicity of the fungus *Diaporthe helianthi* on the leaves of *A. adenophora* [31]. However, *Colletotrichum* may also as an endophyte of *A. adenophora* to resist herbivores, for example, Fang K [31] inoculated *Collectotrichum* into *A. adenophora* to enhance resistance herbivores. Therefore, the role of *Colletotrichum* in *A. adenophora* invasion or its interaction with fungal biocontrol agents is very complex and needs more investigation.

In conclusion, our study reported the diversity of *Colletotrichum* in the leaves of *A. adenophora* and several surrounding crops in the introduced range in China. There were several significant agronomic findings. Firstly, there is a potential application for controlling weeds in agriculture, although it remains to be clarified whether these endophytic *Colletotrichum* species interact with chemical herbicides. The fact that *Colletotrichum* sp. can enhance the pathogenicity of the fungus *D. helianthi* on the leaves of *A. adenophora* [31] provides a potential application for biocontrol of *A. adenophora* by combination of *Colletotrichum* with pathogenic fungi, such as *D. helianthi*. Secondly, because the *Colletotrichum* genus is one of the most important pathogenic fungi [33], our finding highlighted the high disease risk driven by invasive plants in the invaded ecosystem. Indeed, some of these *Colletotrichum* species were previously verified to be pathogenic on local native plants [32], as well as on tested fruits, including grape and mango, as the recent research by Zheng et al. [82] also revealed. This result suggested that invasive species can facilitate pathogen emergence and amplification, raising concerns about the movement of pathogens among agricultural, horticultural and wild grasses. For example, in all, 39 *C. gloeosporioides* and 3 *C. acutatum* isolates were recovered from diseased strawberry crowns, and 52 *C. gloeosporioides* and one *C. acutatum* isolate were recovered from non-cultivated hosts. The results were not inconsistent with the hypothesis that *C. gloeosporioides* isolates obtained from strawberry and noncultivated hosts adjacent to strawberry fields are from the same population and that noncultivated hosts can serve as potential inoculum sources for *Colletotrichum* crown rot of strawberry [83]. Finally, 14 novel *Colletotrichum* species were reported in invasive plants *A. adenophora* and several surrounding crops in south-western China. This result suggested that exotic plants are sources for novel fungal species, and the fungi associated with invasive plants are worth investigating in the future.

## Data Availability

All sequence data are available in NCBI GenBank following the accession numbers in the manuscript.

## Supplementary Materials

The following supporting information can be downloaded at: https://www.mdpi.com/article/10.3390/jof8020185/s1. Table S1: Previously described *Colletotrichum* species and New species used in phylogenetic analyses.
