# Supplementary material for: Fourteen New Species of Foliar Colletotrichum Associated with the Invasive Plant Ageratina adenophora and Surrounding Crops
Source: J Fungi (Basel). 2022 Feb 13;8(2):185. doi: 10.3390/jof8020185 (PMC8879954; doi:10.3390/jof8020185)
Supplement: Supplementary file 1 [file jof-08-00185-s001.zip › Supplementary Table 1.pdf]

Table S1. Previously described *Colletotrichum* species and New species used in phylogenetic analyses

Complex	Species	Strain	Locality	Substrate	ITS	gapdh	chs-1	act	tub2
	C. abscissum	COAD 1877	Brazil	*Citrus sinensis* var. *Valencia*	KP843126	KP843129	KP843132	KP843141	KP843135
	C. acerbum	CBS 128530	New Zealand	*Malus domestica*	JQ948459	JQ948790	JQ949120	JQ949780	JQ950110
	C. acutatum	CBS 112996	Australia	*Grevillea* sp. (a wide range of hosts)	JQ005776	JQ948677	JQ005797	JQ005839	JQ005860
	C. arboricola	CBS 144795	Chile	*Fuchsia magellanica*	MH817944	MH817950	-	MH817956	MH817962
	C. australe	CBS 116478	Australia, South Africa	*Hakea* sp.	JQ948455	JQ948786	JQ949116	JQ949776	JQ950106
**acutatum**	*C. brisbanense*	CBS 292.67	Australia	*Capsicum annuum*	JQ948291	JQ948621	JQ948952	JQ949612	JQ949942
	C. cairnsense	BRIP 63642	Australia	*Capsicum annuum*	KU923672	KU923704	KU923710	KU923716	KU923688
	C. carthami	SAPA100011	Japan	*Carthamus tinctorius*	AB696998	-	-	-	AB696992
	C. chrysanthemi	IMI 364540	China	*Chrysanthemum coronarium*	JQ948273	JQ948603	JQ948934	JQ949594	JQ949924
	C. citri	CBS 134233	China,Yunnan province, Ruili	*Citrus aurantifolia*	KC293581	KC293741	KY856138	KY855973	KC293661

Complex	Species	Strain	Locality	Substrate	ITS	gapdh	chs-1	act	tub2
(continued)	*C. citri*	YMF 1.07322	China,Yunnan province	*Ageratina adenophora*	OK030889	OK513690	OK513587	-	OK513653
	C. cosmi	CBS 853.73	Netherlands	*Cosmos* sp.	JQ948274	JQ948604	JQ948935	JQ949595	JQ949925
	C. cosmi	YMF1.07337	China,Yunnan province	*Ageratina adenophora*	OK030890	OK513691	-	OK513622	-
	C. costaricense	CBS 330.75	Costa Rica	*Coffea* sp.	JQ948180	JQ948510	JQ948841	JQ949501	JQ949831
	C. cuscutae	IMI 304802	Dominica	*Cuscuta* sp.	JQ948195	JQ948525	JQ948856	JQ949516	JQ949846
	C. fioriniae	CBS 128517	Australia, Netherlands, UK, USA	*Magnifera indica* (stem endophyte)	JQ948292	JQ948622	JQ948953	JQ949613	JQ949943
	C. fioriniae	YMF 1.07323	China,Yunnan province	*Ageratina adenophora*	OK030891	OK513692	OK513588	-	-
	C. godetiae	CBS 133.44	Austria, Denmark	*Aeschynomene virginica*	JQ948402	JQ948733	JQ949063	JQ949723	JQ950053
	C. godetiae	YMF1.07330	China,Yunnan province	*Ageratina adenophora*	OK030902	OK513703	OK513598	OK513628	-
	C. guajavae	IMI 350839	India	fruit of *Psidium guajava*	JQ948270	JQ948600	JQ948931	JQ949591	JQ949921
	C. guajavae	YMF1.07334	China,Yunnan province	*Ageratina adenophora*	OK030896	OK513697	-	OK513627	-
	C. indonesiense	CBS 127551	Indonesia	*Eucalyptus* sp.	JQ948288	JQ948618	JQ948949	JQ949609	JQ949939
	C. javanense	CBS 144963a	Indonesia	*Capsicum annuum*	MH846576	MH846572	MH846573	MH846575	MH846574
	C. johnstonii	CBS 128532	New Zealand	*Citrus* sp. (fruit rot)	JQ948444	JQ948775	JQ949105	JQ949765	JQ950095

Complex	Species	Strain	Locality	Substrate	ITS	gapdh	chs-1	act	tub2
(continued)	*C. kinghornii*	CBS 198.35	UK	*Phormium* sp.	JQ948454	JQ948785	JQ949115	JQ949775	JQ950105
	C. laticiphilum	CBS 112989	Inida	*Hevea brasiliensis*	JQ948289	JQ948619	JQ948950	JQ949610	JQ949940
	C. lauri	MFLUCC 17-0205	Italy	*Laurus nobilis*	KY514347	KY514344	KY514341	KY514338	KY514350
	C. limetticola	CBS 114.14	Cuba, USA	*Citrus aurantifolia*	JQ948193	JQ948523	JQ948854	JQ949514	JQ949844
	C. lupine	CBS 109225	Germany	*Camellia* sp.	JQ948155	JQ948485	JQ948816	JQ949476	JQ949806
	C. melonis	CBS 159.84	Brazil	*Cucumis melo*	JQ948194	JQ948524	JQ948855	JQ949515	JQ949845
	C. nymphaeae	CBS 515.78	Netherlands	*Anemone* sp.	JQ948197	JQ948527	JQ948858	JQ949518	JQ949848
	C. nymphaeae	YMF1.07335	China,Yunnan province	*Ageratina adenophora*	OK030899	OK513700	OK513595	-	-
	C. paranaense	CBS 134729	Brazil	*Caryocar brasiliense*	KC204992	KC205026	KC205043	KC205077	KC205060
	C. paxtonii	IMI 165753	St. Lucia (West Indies)	*Musa* sp.	JQ948285	JQ948615	JQ948946	JQ949606	JQ949936
	C. phormii	CBS 118194	Germany	*Phormium* sp.	JQ948446	JQ948777	JQ949107	JQ949767	JQ950097
	C. pyricola	CBS 128531	New Zealand	*Pyrus communis*	JQ948445	JQ948776	JQ949106	JQ949766	JQ950096
	C. rhombiforme	CBS 129953	Portugal, USA	*Olea europaea* (fruit rot)	JQ948457	JQ948788	JQ949118	JQ949778	JQ950108
	C. roseum	CBS 145754	Biobío	*Lapageria rosea*	MK903611	MK903603	-	MK903604	MK903607

Complex	Species	Strain	Locality	Substrate	ITS	gapdh	chs-1	act	tub2
(continued)	*C. salicis*	CBS 607.94	Netherlands	*Acer platanoides*	JQ948460	JQ948791	JQ949121	JQ949781	JQ950111
	C. scovillei	CBS 126529	Indonesia	*Capsicum annuum*	JQ948267	JQ948597	JQ948928	JQ949588	JQ949918
	C. simmondsii	CBS 122122	Australia	*Actinidia chinensis*	JQ948276	JQ948606	JQ948937	JQ949597	JQ949927
	C. simulanticitri	YMF1.07302	China, Yunnan province, Dali county	*Betula* spp.	OK030878	OK513680	OK513577	OK513615	-
	C. simulanticitri	YMF1.07312	China, Yunnan province	*Ageratina adenophora*	OK030879	OK513681	OK513578	OK513616	-
	C. simulanticitri	YMF1.07308	China, Yunnan province	*Ageratina adenophora*	OK030880	OK513682	OK513579	OK513617	-
	C. sloanei	IMI 364297	Malaysia	*Theobroma cacao*	JQ948287	JQ948617	JQ948948	JQ949608	JQ949938
	C. speciosum	YMF1.07301	China, Yunnan province, Simao county	*Ageratina adenophora*	OK030881	-	-	-	-
	C. tamarilloi	CBS 129814	Colombia	*Solanum betaceum*	JQ948184	JQ948514	JQ948845	JQ949505	JQ949835
	C. walleri	CBS 125472	Vietnam	*Coffea* sp.	JQ948275	JQ948605	JQ948936	JQ949596	JQ949926

Complex	Species	Strain	Locality	Substrate	ITS	gapdh	chs-1	act	tub2
	C. wanningense	CGMCC 3.18936	China, Hainan province	*Hevea brasiliensis*	MG830462	MG830318	MG830302	MG830270	MG830286
**agaves**	*C. agaves*	CBS 118190	Mexico, Netherlands	*Agave* spp.	DQ286221	-	-	-	-
	C. ledebouriae	CBS 141284	South Africa	*Ledebouria floridunda*	KX228254	-	-	KX228357	-
	C. sansevieriae	MAFF 239721	Japan	*Sansevieria* sp.	AB212991	-	-	-	-
**boninense**	*C. beeveri*	CBS 128527	New Zealand	*Brachyglottis repanda*	JQ005171	JQ005258	JQ005345	JQ005519	JQ005605
	C. boninense	CBS 123755	Worldwide	*Annonaceae* (a wide range of hosts)	JQ005153	JQ005240	JQ005327	JQ005501	JQ005588
	C. boninense	YMF1.05227	China,Yunnan province	*Ageratina adenophora*	OK030888	OK513689	OK513586	-	OK513652
	C. boninense	YMF1.07311	China,Yunnan province	*Ageratina adenophora*	OK030887	OK513688	OK513585	-	OK513651
	C. brassicicola	CBS 101059	New Zealand	*Brassica oleracea*	JQ005172	JQ005259	JQ005346	JQ005520	JQ005606
	C. colombiense	CBS 129818	Colombia	*Passiflora edulis*	JQ005174	JQ005261	JQ005348	JQ005522	JQ005608
	C. karstii	CORCG6	China, Guizhou Province	*Annona cherimola* (wide range)	HM585409	HM585391	HM582023	HM581995	HM585428
	C. karstii	YMF1.07306	China,Yunnan province	*Ageratina adenophora*	OK030897	OK513698	OK513593	-	OK513658

Complex	Species	Strain	Locality	Substrate	ITS	gapdh	chs-1	act	tub2
	C. karstii	YMF1.07307	China,Yunnan province	*Ageratina adenophora*	OK030898	OK513699	OK513594	-	OK513659
**dematium**	*C. dematium*	CBS 125.25	France	*Bidens pilosa*	GU227819	GU228211	GU228309	GU227917	GU228113
	C. eryngiicola	MFLUCC 17-0318	Russia	*Eryngium campestre*	KY792726	KY792723	KY792720	KY792717	KY792729
	C. orchidis	MFLUCC 17-1302	Italy	*Orchis* sp.	MK502144	MK496857	MK496855	MK496853	MK496859
**destructivum**	*C. americae-borealis*	CBS 136232	USA	*Medicago sativa*	KM105224	KM105579	KM105294	KM105434	KM105504
	C. destructivum	CBS 136228	Worldwide	genera of *Asteraceae*	KM105207	KM105561	KM105277	KM105417	KM105487
	C. lini	CBS 172.51	Netherlands	*Linum* sp.	JQ005765	KM105581	JQ005786	JQ005828	JQ005849
	C. ocimi	CBS 298.94	Italy,Australia	*Ocimum basilicum*	KM105222	KM105577	KM105292	KM105432	KM105502
**dracaenophilum**	*C. cariniferi*	MFLUCC 14-0100	Thailand	*Dendrobium cariniferum*	MF448521	-	-	-	MH351274
	C. coelogynes	CBS 132504	Germany	*Coelogyne* sp.	MG600713	MG600776	MG600836	MG600920	MG600980
	C. dracaenophilum	CBS 118199	Bulgaria	*Dracaena* sp.	JX519222	JX546707	JX519230	JX519238	JX519247
	C. excelsum-altitudinum	CGMCC 3.15130	China	*Bletilla ochracea*	HM751815	KC843502	-	KC843548	JX625211
	C. parallelophorum	MFLUCC 14-0083	Thailand	*Dendrobium* sp.	MF448525	MK165695	-	-	MH351280
	C. robustum	YMF1.06941	China, Guizhou province, Dushan county	*Ageratina adenophora*	OK030877	-	OK513576	OK513614	OK513646

Complex	Species	Strain	Locality	Substrate	ITS	gapdh	chs-1	act	tub2
	C. tongrenense	GMBC0209	China, Guizhou province, Tongren	*Nothapodytes pittosporoides*	MH482933	MH705332	-	MH717074	MH729805
	C. tropicicola	MFLUCC 11-0114	Thailand	*Citrus maxima*	JN050240	JN050229	-	JN050218	JN050246
	C. yunnanense	CBS 13213	China, Yunnan, Kunming	*Buxus* sp.	JX546804	JX546706	JX519231	JX519239	JX519248
**gigasporum**	*C. gigasporum*	CBS 133266	Madagascar	*Camelia sinensis*	KF687715	KF687822	KF687761	-	KF687866
	C. gigasporum	YMF1.07331	China,Yunnan province	*Ageratina adenophora*	OK030895	OK513696	OK513592	OK513626	OK513657
	C. gigasporum	YMF1.07336	China,Yunnan province	*Ageratina adenophora*	OK030894	OK513695	OK513591	OK513625	OK513656
	C. pseudomajus	CBS 571.88	Taiwan	*Camellia sinensis*	KF687722	KF687826	KF687779	KF687801	KF687883
	C. aenigma	ICMP 18608	Israel	*Persea americana*	JX010244	JX010044	JX009774	JX009443	JX010389
	C. aeschynomenes	ICMP 17673	USA	*Aeschynomene virginica*	JX010176	JX009930	JX009799	JX009483	JX010392
	C. alatae	CBS 304.67	Barbardos, India	*Dioscorea alata*	JX010190	JX009990	JX009837	JX009471	JX010383
	C. alienum	ICMP 12071	New Zealand	*Banksia dryandroides*	JX010251	JX010028	JX009882	JX009572	JX010411

Complex	Species	Strain	Locality	Substrate	ITS	gapdh	chs-1	act	tub2
**gloeosporioides**	*C. analogum*	YMF1.06943	China, Yunnan province, Ning'er county	*Ageratina adenophora*	OK030860	OK513663	OK513559	OK513599	OK513629
	C. analogum	YMF1.07327	China, Yunnan province	*Ageratina adenophora*	OK030861	OK513664	OK513560	OK513600	OK513630
	C. analogum	YMF1.07304	China, Yunnan province	*Ageratina adenophora*	OK030862	OK513665	OK513561	OK513601	OK513631
	C. aotearoa	ICMP 18537	New Zealand	*Banksia marginata*	JX010205	JX010005	JX009853	JX009854	JX010420
	C. arecicola	CGMCC 3.19667	China	*Areca catechu*	MK914635	MK945455	MK935541	MK935374	MK935498
	C. artocarpicola	MFLUCC 18-1167	Thailand	*Artocarpus heterophyllus*	MN415991	MN435568	MN435569	MN435570	MN435567
	C. asianum	ICMP 18580	Thailand	*Coffea arabica*	JX010196	JX010053	JX009867	JX009584	JX010406
	C. camelliae	CGMCC 3.14925	China, Guizhou Province	*Camellia sinensis*	KJ955081	KJ954782	-	KJ954363	KJ955230
	C. cangyuanensis	YMF1.05000	China, Yunnan province, Cangyuan county	*Ageratina adenophora*	OK030863	OK513666	OK513562	OK513602	OK513632

Complex	Species	Strain	Locality	Substrate	ITS	gapdh	chs-1	act	tub2
	C. cangyuanensis	YMF1.05001	China, Yunnan province	*Ageratina adenophora*	OK030864	OK513667	OK513563	OK513603	OK513633
	C. cangyuanensis	YMF1.04998	China, Yunnan province	*Ageratina adenophora*	OK030865	OK513668	OK513564	OK513604	OK513634
	C. changpingense	MFLUCC 15-0022	China, Beijing City, Changping	*Fragaria × ananassa*	KP683152	KP852469	KP852449	KP683093	KP852490
	C. chrysophillum	CMM4268	Brazil	*Musa* sp.	KX094252	KX094183	KX094083	KX093982	KX094285
	C. ciggaro	ICMP 18539	Australia	*Olea europaea*	JX010230	JX009966	JX009800	JX009523	JX010434
	C. clidemiae	ICMP 18658	USA	*Clidemia hirta*	JX010265	JX009989	JX009877	JX009537	JX010438
(continued)	*C. cobbittiense*	BRIP 66219a	Australia	*Cordyline* sp.	MH087016	MH094133	MH094135	MH094134	MH094137
	C. conoides	CAUG17*	China, Jiangsu Province, Nanjing City	*Capsicum annuum*	KP890168	KP890162	KP890156	KP890144	KP890174
	C. cordylinicola	ICMP 18579	Laos	*Eugenia javanica*	JX010226	JX009975	JX009864	HM470234	JX010440
	C. dimorphum	YMF1.07303	China, Guizhou province, Pingtang county	*Ageratina adenophora*	OK030866	OK513669	OK513565	OK513605	OK513635

Complex	Species	Strain	Locality	Substrate	ITS	gapdh	chs-1	act	tub2
(continued)	*C. dimorphum*	YMF1.07309	China, Guizhou province, Pingtang county	*Ageratina adenophora*	OK030867	OK513670	OK513566	OK513606	OK513636
	C. endophytica	MFLUCC 13–0418	Thailand	*Pennisetum purpureum*	KC633854	KC832854	-	KF306258	-
	C. fructicola	ICMP 18581	worldwide	wide range	JX010165	JX010033	JX009866	FJ907426	JX010405
	C. fructicola	YMF1.07305	China,Yunnan province	*Ageratina adenophora*	OK030893	OK513694	OK513590	OK513624	OK513655
	C. fructicola	YMF1.07325	China,Yunnan province	*Ageratina adenophora*	OK030892	OK513693	OK513589	OK513623	OK513654
	C. fructivorum	CBS 133125	Canada, Colombia, USA	*Vaccinium macrocarpon*	JX145145	-	-	-	JX145196
	C. gloeosporioides	CBS 112999	Australia	*Citrus* sp.	JQ005152	JQ005239	JQ005326	JQ005500	JQ005587
	C. gracile	YMF1.06939	China, Guizhou province, Nayong county	*Ageratina adenophora*	OK030868	OK513671	OK513567	OK513607	OK513637
	C. gracile	YMF1.07329	China, Yunnan province	*Ageratina adenophora*	OK030869	OK513672	OK513568	OK513608	OK513638
	C. grevilleae	CBS 132879	Italy	*Grevillea* sp.	KC297078	KC297010	KC296987	KC296941	KC297102

Complex	Species	Strain	Locality	Substrate	ITS	gapdh	chs-1	act	tub2
	C. grossum	CAUG7*	China, Hainan Province	*Capsicum annuum*	KP890165	KP890159	KP890153	KP890141	KP890171
	C. hebeiense	MFLUCC 13–0726	China, Hebei province	*Vitis vinifera*	KF156863	KF377495	KF289008	KF377532	KF288975
	C. hederiicola	MFLU 15-0689	Italy	*Hedera helix*	MN631384	-	MN635794	MN635795	-
	C. helleniense	CBS 142418	Greece	*Citrus reticulata*	KY856446	KY856270	KY856186	KY856019	KY856528
	C. henanense	CGMCC 3.17354	China, Henan Province, Xinyang	*Camellia sinensis*	KJ955109	KJ954810	-	KM023257	KJ955257
(continued)	*C. horii*	ICMP 10492	Brazil, China	*Diospyros kaki*	GQ329690	GQ329681	JX009752	JX009438	JX010450
	C. hystricis	CBS 142411	Italy	*Citrus hystrix*	KY856450	KY856274	KY856190	KY856023	KY856532
	C. jiangxiense	CGMCC 3.17363	China, Jiangxi Province, Ganzhou	*Camellia sinensis*	KJ955201	KJ954902	-	KJ954471	KJ955348
	C. kahawae	ICMP17816	African continent	*Coffea arabica*	JX010231	JX010012	JX009813	JX009452	JX010444
	C. makassarense	CBS 143664a	Indonesia	*Capsicum annuum*	MH728812	MH728820	MH805850	MH781480	MH846563
	C. musae	ICMP19119	Worldwide	*Musa* sp.	HQ596292	HQ596299	JX009896	HQ596284	HQ596280
	C. nanhuaensis	YMF1.04993	China, Yunnan province, Nanhua county	*Ageratina adenophora*	OK030870	OK513673	OK513569	OK513609	OK513639

Complex	Species	Strain	Locality	Substrate	ITS	gapdh	chs-1	act	tub2
	C. nanhuaensis	YMF1.04990	China, Yunnan province	*Ageratina adenophora*	OK030871	OK513674	OK513570	OK513610	OK513640
	C. nullisetosum	YMF1.06946	China, Yunnan province, ning'er county	*Mangifera indica*	OK030872	OK513675	OK513571	OK513611	OK513641
	C. nullisetosum	YMF1.07328	China, Yunnan province	*Mangifera indica*	OK030873	OK513676	OK513572	OK513612	OK513642
(continued)	*C. nupharicola*	ICMP 18187	USA	*Nuphar, Nymphae*	JX010187	JX009972	JX009835	JX009437	JX010398
	C. oblongisporum	YMF1.06938	China, Yunnan province, Kunming city	*Ageratina adenophora*	OK030874	OK513677	OK513573	-	OK513643
	C. oblongisporum	YMF1.07326	China, Yunnan province	*Ageratina adenophora*	OK030875	OK513678	OK513574	-	OK513644
	C. pandanicola	MFLUCC 17-0571	Thailand	*Pandanus* sp.	MG646967	MG646934	MG646931	MG646938	MG646926
	C. parvisporum	YMF1.06942	China, Guangxi province, Debao	*Ageratina adenophora*	OK030876	OK513679	OK513575	OK513613	OK513645

Complex	Species	Strain	Locality	Substrate	ITS	gapdh	chs-1	act	tub2
	C. perseae	CBS 141365	Israel	*Persea Americana*	KX620308	KX620242	-	KX620145	KX620341
	C. pseudotheobromicola	MFLUCC 18–1602	China, Beijing	*Prunus avium*	MH817395	MH853675	MH853678	MH853681	MH853684
	C. psidii	CBS 145.29	Italy	*Psidium* sp.	JX010219	JX009967	JX009901	JX009515	JX010443
	C. proteae	CBS 132882	South Africa	*Protea* sp.	KC297079	KC297009	KC296986	KC296940	KC297101
	C. queenslandicum	ICMP 1778	Australia	*Anacardium occidentale*	JX010276	JX009934	JX009899	JX009447	JX010414
	C. rhexiae	CBS 133134	USA	*Rhexia virginica*	JX145128	-	-	-	JX145179
	C. salsolae	ICMP 19051	Hungary	*Salsola tragus*	JX010242	JX009916	JX009863	JX009562	JX010403
(continued)	*C. siamense*	ICMP 18578	worldwide	wide range	FJ972613	FJ972575	JX009865	FJ907423	FJ907438
	C. subhenanense	YMF1.06865	China, Yunnan province, Cangyuan county	*Ageratina adenophora*	OK030883	OK513684	OK513581	OK513618	OK513647
	C. subhenanense	YMF1.07324	China, Yunnan province	*Ageratina adenophora*	OK030884	OK513685	OK513582	OK513619	OK513648
	C. syzygiicola	MFLUCC 10-0624	Thailand	*Citrus aurantifolia*	KF242094	KF242156	-	KF157801	KF254880
	C. tainanense	CBS 143666a	Taiwan	-	MH728818	MH728823	MH805845	MH781475	MH846558
	C. theobromicola	ICMP 18649	worldwide	wide range of hosts	JX010294	JX010006	JX009869	JX009444	JX010447

Complex	Species	Strain	Locality	Substrate	ITS	gapdh	chs-1	act	tub2
(continued)	*C. temperatum*	CBS 133122	USA	*Vaccinium macrocarpon*	JX145159	-	-	-	JX145211
	C. ti	ICMP 4832	New Zealand	*Cordyline* sp.	JX010269	JX009952	JX009898	JX009520	JX010442
	C. tropicale	CBS 124949	tropical region	wide host range	JX010264	JX010007	JX009870	JX009489	JX010407
	C. viniferum	GZAAS5.08601	China,Yunnan Province	*Vitis vinifera*	JN412804	JN412798	-	JN412795	JN412813
	C. wuxiense	CGMCC 3.17894	China, Jiangsu Province	*Camellia sinensis*	KU251591	KU252045	KU251939	KU251672	KU252200
	C. xanthorrhoeae	ICMP 17903	Australia	*Xanthorrhoea preissii*	JX010261	JX009927	JX009823	JX009478	JX010448
	C. yulongense	CFCC 50818	China,Yunnan Province	*Vaccinium dunalianum*	MH751507	MK108986	MH793605	MH777394	MK108987
	C. yunajiangensis	YMF1.04996	China, Yunnan province, yuanjiang county	*Ageratina adenophora*	OK030885	OK513686	OK513583	OK513620	OK513649
	C. yunajiangensis	YMF1.04997	China, Yunnan province	*Ageratina adenophora*	OK030886	OK513687	OK513584	OK513621	OK513650
	C. alcornii	IMI176619	Australia	*Bothriochloa bladhii*	JX076858	-	-	-	-
	C. axonopodi	IMI 279189	Australia	*Axonopus* spp.	MN521699	-	-	-	-

Complex	Species	Strain	Locality	Substrate	ITS	gapdh	chs-1	act	tub2
**graminicola-caudatum**	*C. baltimorense*	BPI892771	USA	*Sorghastrum nutans*	JX076866	-	-	-	-
	C. caudatum	CBS 131602	USA	*Sorghastrum nutans*	JX076860	-	-	-	-
	C. caudasporum	CGMCC 3.15106	China	*Bletilla ochracea*	JX625162	KC843512	-	KC843526	JX625190
	C. duyunensis	CGMCC 3.15105	China	*Bletilla ochracea*	JX625160	KC843515	-	KC843530	JX625187
	C. echinochloae	MAFF 511473	Japan	*Echinochloa* sp.	AB439811	-	-	-	-
	C. eleusines	MAFF 511155	Japan, USA	*Elusines* sp.	JX519218	-	JX519226	JX519234	JX519243
	C. endophytum	CGMCC 3.15108	China	*Bletilla ochracea*	JX625177	KC843521	-	KC843533	JX625206
	C. eremochloae	CBS 129661	China, USA	*Eremochloae ophiuroides*	JX519220	-	JX519228	JX519236	JX519245
	C. falcatum	CBS 147945	Australia, Indonesia	*Saccharum officinarum*	JQ005772	-	JQ005793	JQ005835	JQ005856
	C. graminicola	M 1.001/CBS 130836	Worldwide	*Zea mays*	JQ005767	-	JQ005788	JQ005830	JQ005851
	C. hanaui	MAFF 305404	China, Japan, USA	*Digitaria ciliaris*	JX519217	-	JX519225	-	JX519242
	C. jacksonii	MAFF 305460	Japan	*Echinochloa esculenta*	JX519216	-	JX519224	JX519233	JX519241
	C. miscanthi	MAFF 510857	Japan	*Miscanthus sinensis*	JX519221	-	JX519229	JX519237	JX519246
	C. navitas	CBS 125086	USA	*Panicum* sp.	JQ005769	-	JQ005790	JQ005832	JQ005853
	C. nicholsonii	MAFF 511115	Japan, New Zealand	*Paspalum dilatatum*	JQ005770	-	JQ005791	JQ005833	JQ005854

Complex	Species	Strain	Locality	Substrate	ITS	gapdh	chs-1	act	tub2
	C. ochracea	CGMCC 3.15104	China,Guizhou Province	*Bletilla ochracea*	JX625168	KC843513	-	KC843527	JX625183
	C. paspali	MAFF 305403	Japan	*Paspalum notatum*	JX519219	-	JX519227	JX519235	JX519244
	C. somersetense	CBS 131599	USA	*Sorghastrum nutans*	JX076862	-	-	-	-
	C. sublineola	CBS 131301, S3.001	Togo, USA	*Sorghum* sp.	JQ005771	-	JQ005792	JQ005834	JQ005855
	C. sp.	YMF1.06940	China, GuiZhou province, Nayong county	*Ageratina adenophora*	OK030882	OK513683	OK513580	-	-
	C. zoysiae	MAFF238573	Japan	*Zoysia tenuifolia*	JX076871	-	-	-	-
**magnum**	*C. liaoningense*	CGMCC 3.17616	China,Liaoning Province	*Capsicum annuum*	KP890104	KP890135	KP890127	KP890097	KP890111
	C. magnum	CBS 519.97	USA	*Citrullus lanatus*	MG600769	MG600829	MG600875	MG600973	MG601036
	C. okinawense	MAFF 240517	Japan Brazil	*Carica papaya*	MG600767	MG600827	-	MG600971	MG601034
**orbiculare**	*C. orbiculare*	CBS 570.97	Worldwide	genera of *Cucubitaceae*	KF178466	KF178490	KF178515	KF178563	KF178587
	C. sidae	CBS 504.97	USA	*Sida spinosa*	KF178472	KF178497	KF178521	KF178569	KF178593

Complex	Species	Strain	Locality	Substrate	ITS	gapdh	chs-1	act	tub2
	C. spinosum	CBS 515.97	Argentina, Australia	*Xanthium spinosum*	KF178474	KF178498	KF178523	KF178571	KF178595
	C. tebeesti	CBS 522.97	Canada	*Malva pusilla*	KF178473	KF178505	KF178522	KF178570	KF178594
orchidearum	*C. cattleyicola*	CBS 170.49	Belgium, Japan	*Cattleya* sp.	MG600758	MG600819	MG600866	MG600963	MG601025
	C. cliviicola	CBS 125375	China,Yunnan Province	*Clivia* sp.	MG600733	MG600795	MG600850	MG600939	MG601000
	C. musicola	CBS 132885	Mexico	*Musa* sp.	MG600736	MG600798	MG600853	MG600942	MG601003
	C. orchidearum	CBS 135131	China, Iran	*Cordyline*	MG600738	MG600800	MG600855	MG600944	MG601005
	C. piperis	IMI 71397	Malaysia	*Piper nigrum*	MG600760	MG600820	MG600867	MG600964	MG601027
	C. plurivorum	CBS 125474	Benin, Brazil, China, Iran	*Abelmoschus esculentus*	MG600718	MG600781	MG600841	MG600925	MG600985
	C. plurivorum	YMF1.07332	China,Yunnan province	*Ageratina adenophora*	OK030900	OK513701	OK513596	-	OK513660
	C. plurivorum	YMF1.07333	China,Yunnan province	*Ageratina adenophora*	OK030901	OK513702	OK513597	-	OK513661
	C. sojae	ATCC 62257	Brazil, China, Iran, Italy	*Arctium lappa*	MG600749	MG600810	MG600860	MG600954	MG601016
	C. vittalense	CBS 181.82	India	*Calamus thwaitesii*	MG600734	MG600796	MG600851	MG600940	MG601001
spaethianum	*C. guizhouensis*	CGMCC 3.15112	China,Guizhou Province	*Bletilla ochracea*	JX625158	KC843536	-	KC843536	JX625185
	C. lilii	CBS 109214	Japan	*Lillium* sp.	GU227810	GU228202	GU228300	GU227908	GU228104

Complex	Species	Strain	Locality	Substrate	ITS	gapdh	chs-1	act	tub2
	C. spaethianum	CBS 167.49	Germany	*Hosta sieboldiana*	GU227807	GU228199	GU228297	GU227905	GU228101
**truncatum**	*C. curcumae*	IMI 288937	India	*Curcuma longa*	GU227893	GU228285	GU228383	GU227991	GU228187
	C. jasminigenum	MFLUCC 10-0273	Vietnam	*Jasminum sambac*	HM131513	-	-	-	HM153770
	C. truncatum	CBS 151.35	Australia, Bangladesh	*Arachis hypogaea*	GU227862	GU228254	GU228352	GU227960	GU228156
**singleton**	*C. adenophorae*	YMF1.04952	China, Yunnan province, Lancang county	*Ageratina adenophora*	OK030859	OK513662	OK513558	-	-
	C. citrus-medicae	GUCC 1554	China, Kunming	*Citrus medica*	MN959910	MT006331	MT006328	MT006325	-
	C. chlorophyti	IMI 103806	Australia, India	*Chlorophytum* sp.	GU227894	GU228286	GU228384	GU227992	GU228188
	C. coccodes	CBS 369.75	The Netherlands	*Rubus coreanus*	HM171679	HM171673	JQ005796	HM171667	JQ005859
	C. hsienjenchang	MAFF 243051	Japan	*Phyllostachys* sp.	AB738855	-	AB738846	AB738845	-
	C. metake	MAFF 244029	Japan	*Pleioblastus simoni*	AB738859	-	-	-	-
	C. nigrum	CBS 169.49	Argentina	*Capsicum* sp.	JX546838	JX546742	JX546693	JX546646	JX546885
	C. orchidophilum	CBS 632.80	Panama, UK, USA, France	*Ascocenda* sp.	JQ948151	JQ948481	JQ948812	JQ949472	JQ949802

Complex	Species	Strain	Locality	Substrate	ITS	gapdh	chs-1	act	tub2
	C. phaseolorum	CBS 158.36	Japan	*Phaseolus* sp.	GU227897	GU228289	GU228387	GU227995	GU228191
	C. pseudoacutatum	CBS 436.77	Chile	*Pinus radiata*	JQ948480	JQ948811	JQ949141	JQ949801	JQ950131
	C. pyrifolia	CGMCC 3.18902	China, Hubei Province	*Pyrus pyrifolia*	MG748078	MG747996	MG747914	MG747768	MG748158
	C. rusci	CBS 119206	Italy	*Ruscus* sp.	GU227818	GU228210	GU228308	GU227916	GU228112
	C. sydowii	CBS135819	Taiwan (China)	*Sambucus* sp.	KY263783	KY263785	KY263787	KY263791	KY263793
	C. trichellum	CBS 217.64	Canada, Germany	*Hedera* sp.	GU227812	GU228204	GU228302	GU227910	GU228106
**outgroup**	*Monilochaetes infuscans*	CBS 869.96	District of Columbia	*Ipomoea batata*	JQ005780	JX546612	JQ005801	-	JQ005864
