# Supplementary figures and images for: Fourteen New Species of Foliar Colletotrichum Associated with the Invasive Plant Ageratina adenophora and Surrounding Crops
Source: J Fungi (Basel). 2022 Feb 13;8(2):185. doi: 10.3390/jof8020185 (PMC8879954; doi:10.3390/jof8020185)

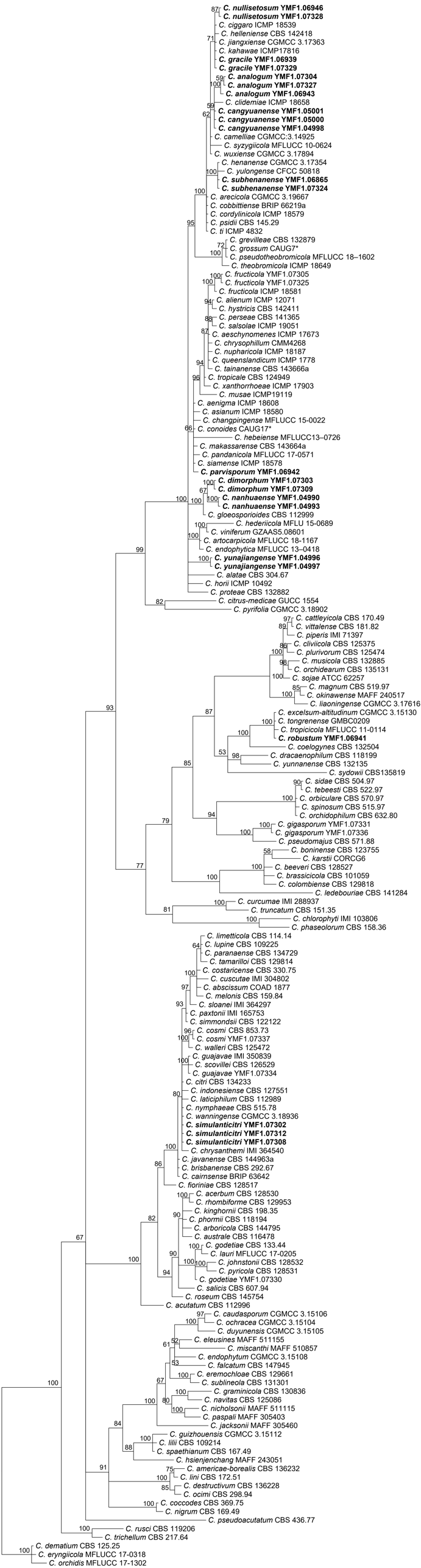

Supplement: Supplementary file 1 [file jof-08-00185-s001.zip › Phylogenic tree-ACT.pdf]

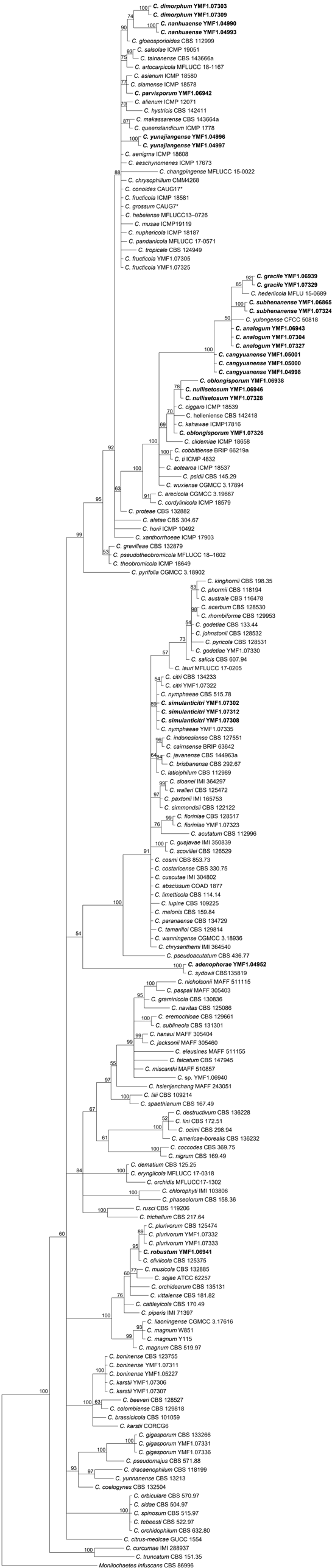

Supplement: Supplementary file 1 [file jof-08-00185-s001.zip › Phylogenic tree-CHS.pdf]

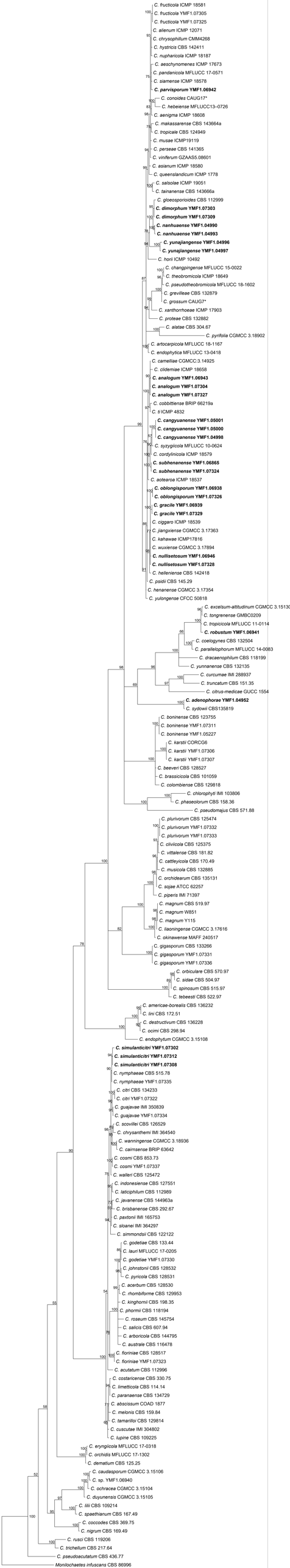

Supplement: Supplementary file 1 [file jof-08-00185-s001.zip › Phylogenic tree-GAPDH.pdf]

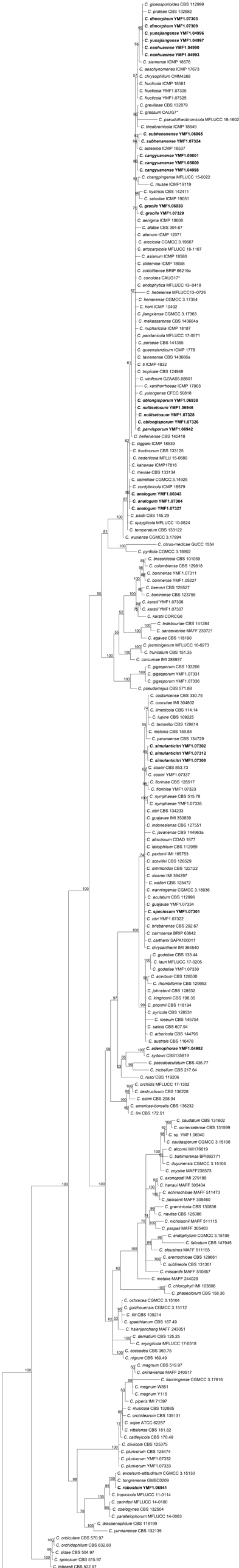

Supplement: Supplementary file 1 [file jof-08-00185-s001.zip › Phylogenic tree-ITS.pdf]

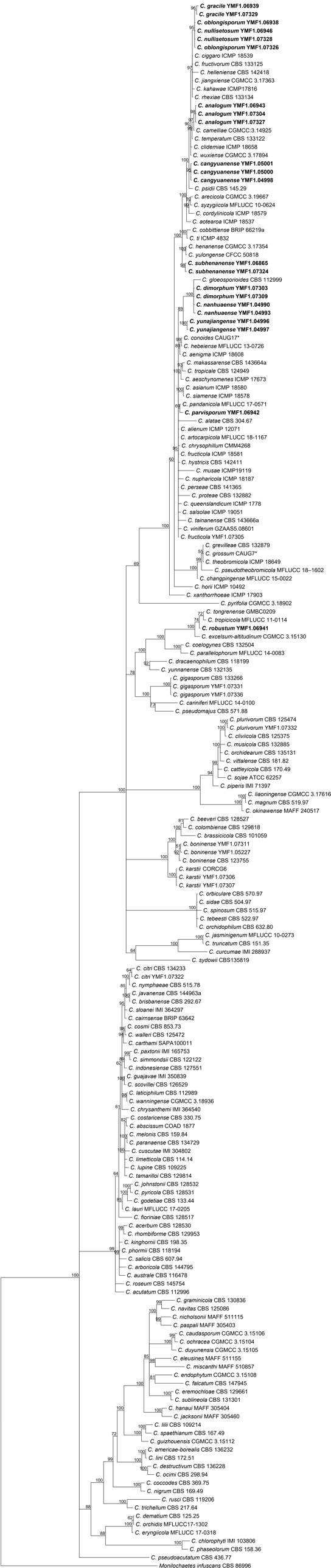

Supplement: Supplementary file 1 [file jof-08-00185-s001.zip › Phylogenic tree-TUB.pdf]
